# Multi-omics association study of DNA methylation and gene expression levels and diagnoses of cardiovascular diseases in Danish Twins

**DOI:** 10.1186/s13148-024-01727-6

**Published:** 2024-08-26

**Authors:** Asmus Cosmos Skovgaard, Afsaneh Mohammadnejad, Hans Christian Beck, Qihua Tan, Mette Soerensen

**Affiliations:** 1 The Danish Twin Registry and the Research Unit for Epidemiology, Biostatistics and Biodemography, Department of Public Health, University of Southern Denmark, Campusvej 55, 5230 Odense M, Denmark; 2 Center for Individualized Medicine in Arterial Diseases, Department of Biochemistry, Odense University Hospital, J.B. Winsloews Vej 4, 5000 Odense C, Denmark; 3 Department of Clinical Genetics, Odense University Hospital, J.B. Winsloews Vej 4, 5000 Odense C, Denmark

**Keywords:** Multi-omics association study, Twins, Cardiovascular diseases, Response pathways versus regulatory pathways, *MYEO1E* gene, *DDX5* gene, Gene set enrichment, Interaction networks

## Abstract

**Background:**

Cardiovascular diseases (CVDs) are major causes of mortality and morbidity worldwide; yet the understanding of their molecular basis is incomplete. Multi-omics studies have significant potential to uncover these mechanisms, but such studies are challenged by genetic and environmental confounding—a problem that can be effectively reduced by investigating intrapair differences in twins. Here, we linked data on all diagnoses of the circulatory system from the nationwide Danish Patient Registry (spanning 1977–2022) to a study population of 835 twins holding genome-wide DNA methylation and gene expression data. CVD diagnoses were divided into prevalent or incident cases (i.e., occurring before or after blood sample collection (2007–2011)). The diagnoses were classified into four groups: cerebrovascular diseases, coronary artery disease (CAD), arterial and other cardiovascular diseases (AOCDs), and diseases of the veins and lymphatic system. Statistical analyses were performed by linear (prevalent cases) or cox (incident cases) regression analyses at both the individual-level and twin pair-level. Significant genes (*p* < 0.05) in both types of biological data and at both levels were inspected by bioinformatic analyses, including gene set enrichment analysis and interaction network analysis.

**Results:**

In general, more genes were found for prevalent than for incident cases, and bioinformatic analyses primarily found pathways of the immune system, signal transduction and diseases for prevalent cases, and pathways of cell–cell communication, metabolisms of proteins and RNA, gene expression, and chromatin organization groups for incident cases. This potentially reflects biology related to response to CVD (prevalent cases) and mechanisms related to regulation and development of disease (incident cases). Of specific genes, Myosin 1E was found to be central for CAD, and DEAD-Box Helicase 5 for AOCD. These genes were observed in both the prevalent and the incident analyses, potentially reflecting that their DNA methylation and gene transcription levels change both because of disease (prevalent cases) and prior disease (incident cases).

**Conclusion:**

We present novel biomarkers for CVD by performing multi-omics analysis in twins, hereby lowering the confounding due to shared genetics and early life environment—a study design that is surprisingly rare in the field of CVD, and where additional studies are highly needed.

**Supplementary Information:**

The online version contains supplementary material available at 10.1186/s13148-024-01727-6.

## Background

Globally, cardiovascular diseases (CVDs) remain the leading cause of death, especially deaths due to coronary artery disease (CAD) and stroke are frequent [1]. Overall, CVD comprise several complex heterogeneous diseases of the circulatory system caused by an interplay between biological variation, and physiological, environmental, and lifestyle factors [1]. Although the prevalence of CVD continues to increase globally, a detailed understanding of the molecular mechanisms underlying CVD is still incomplete.

Genome-wide association studies (GWASs) have identified several genetic loci associated with CVD [2–6]. For instance, GWAS of CAD have identified more than 160 genetic loci during the last decade [5], and new studies continue to detect novel loci. For instance, recently 95 loci were identified in 1,093,078 individuals (including 243,392 CAD cases) [6]. Moreover, polygenetic risk scores of CVD have been published, summarizing the risk related to the identified genetic variants [7]. Nevertheless, such GWAS provide little or no molecular evidence of gene causality. This realization led to the integration of genetic data with additional high-throughput molecular data designed to investigate the epigenome, transcriptome, proteome, or metabolome, thus enabling the integrative analysis of multi-omics data to potentially discover casual genes and identify essential molecular mechanisms involved in CVD progression [8].

Epigenetics is a regulatory mechanism that can alter gene activity without changing the DNA sequence, where the most studied epigenetic mechanism is DNA methylation [9]. DNA methylation plays a key role in gene expression and mainly occurs in cytosine contiguous with guanine (CpGs). Several epigenome-wide association studies (EWASs) have identified CpGs showing differential methylation patterns related to CVD [10–12] and its cardiometabolic risk factors such as blood pressure [13], lipid levels [14], and body mass index (BMI) [15]. For instance, a cohort study by Agha *et. al.* identified an increase of methylation of 52 CpGs to be associated with CAD in 11,461 individuals (including 1895 CAD cases) [11]. Transcriptomics, also known as gene expression profiling, refers to the study of gene expression itself as measured by RNA levels [16]. A limited number of transcriptomics-wide association studies (TWASs) have identified genes linked to CVD [17, 18]. For instance, Liao et al*.* showed that 190 mRNAs were upregulated, and 176 mRNAs were downregulated significantly in 40 CAD patients compared to 20 controls [18]. Lastly, the integration of DNA methylation data and gene expression data in relation to CVD is rare. To the best of our knowledge, only two studies have been published on this topic: a recent study by Xiaokang Z. et al*.* [19] found five genes (*ATG7*, *BACH2*, *CDK1B*, *DHCR24*, and *MPO*) that were regulated by DNA methylation and associated with CAD in 2,085 participants. The other study by Palou-Márquez et al*.* [20] identified three independent latent factors (named 9, 21, and 27) linked to CAD: factor 9 related to age- and cell-type proportions; factor 21 related to immunodeficiency virus infection-related pathway and inflammation; and factor 27 related to lifestyle factors like smoking, alcohol consumption, and BMI. The study was performed in 2620 and 1892 participants with DNA methylation and gene expression data, respectively.

However, one challenge in investigating the association between nongenetic biomarkers and CVD is that this association might be confounded by genetic variation. One way to control for this genetic bias is to investigate twin pairs that are discordant for the disease, i.e., where one twin develops the disease of interest, while the co-twin does not. This study design is known as the discordant twin pair design [21], and it is a statistically efficient approach to control for confounding factors [22]. For instance, for DNA methylation array data, it has been estimated that, compared to the classical case–control design, the discordant twin pair design needs down to only one-tenth the individuals for a trait with a heritability of 60% [22]. The discordant twin pair design also controls for differences in environmental factors potentially confounding the association, especially factors related to the shared early life environment. Hence, the aim of the current study was to integrate DNA methylation and gene expression data to detect molecular mechanisms associated with CVD in Danish twins. The integration of several omics layers provides the possibility to investigate the complexity of CVD and potentially reveal new predictive biomarkers and therapeutic targets [23]. To the best of our knowledge, this is the first twin study to integrate DNA methylation and gene expression data to identify molecular mechanisms associated with CVD.

## Materials and methods

**Fig. 1:**
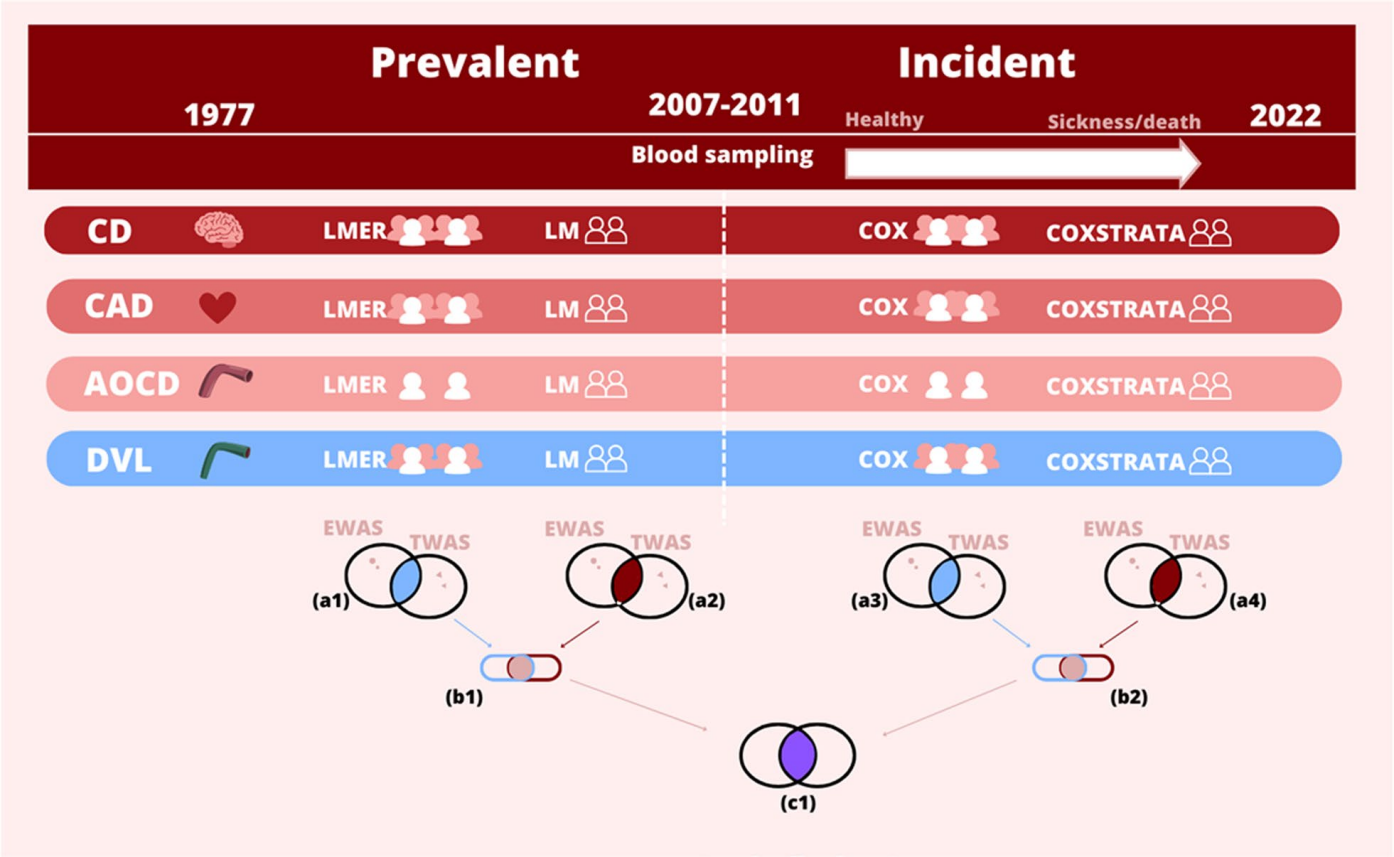
Workflow of the study. Top of figure: disease diagnoses from the Danish Patient Registry spanned 1977–2022, while blood samples were collected in 2007–2011. Diagnoses were divided into prevalent cases (i.e., before blood sampling, left side of figure) and incident cases (i.e., after blood sampling, right side of figure), the latter with cases not having a diagnosis before blood sampling in the disease group of interest. For both prevalent and incident cases, statistical analyses were performed of the epigenetic data (EWAS) and of the gene expression data (TWAS), both at the individual-level and at the twin pair-level (the latter reducing confounding induced by genetics and shared environment). The individual-level analyses are depicted in the figure as individuals in pink and white, while the twin pair-level analyses are depicted as two individuals. Bottom of figure: for each of the four disease groups the following statistical analyses were performed: a1–a4: first, the genes with a *p* value below 0.05 in both analysis of the epigenetic data (EWAS) and in analysis of the gene expression data (TWAS) were found within each test (e.g., LMER depicted in light blue), b1 + b2: then of these overlapping genes, the genes found both at the individual-level and at the twin pair-level were identified (depicted in beige), and lastly, (c1) then of these overlapping genes, the genes found both for prevalent and incident cases were identified (depicted in purple). The b1, b2, and c1 overlaps for each disease group were investigated by gene set enrichment and network interaction analyses, while the overlaps a1–a4 were reported as lists of genes. Abbreviations: (1) disease diagnoses: CD: cerebrovascular diseases, CAD; coronary artery disease, AOCD: arterial and other cardiovascular diseases and DVL: diseases of the veins and lymphatic system (see Sect. “Cardiovascular disease diagnoses from the Danish National Patient Registry” for details), (2) statistical analyses: LMER: linear mixed effect regression analysis (individual-level analysis of prevalent cases), LM: linear regression analysis (twin pair-level analysis of prevalent cases), COX: Cox proportional hazards regression analysis (individual-level analysis of incident cases) and COXSTRATA: stratified Cox proportional hazards regression analysis (twin pair-level analysis of incident cases) (see Sect. “Statistical analyses” for details) and (3) EWAS: epigenome-wide association analysis, and TWAS: transcriptome-wide association analysis

For the present study, genome-wide epigenetic data and genome-wide gene transcription data were available for 835 twins (including 412 complete twin pairs). These data were analyzed together with information on diagnoses of CVD obtained for the same individuals from the Danish National Patient Registry (DNPR) [24]. The study design is depicted in Fig. 1 and explained below, and additional information regarding biological data and data preparation can be found in Supplementary Methods.

### Study population

The study population comprised 835 twins drawn from three population-based and nationwide surveys from the Danish Twin Registry [25]: The Longitudinal Study of Aging Danish Twins (LSADT), The Middle Age Danish Twin study (MADT), and the Birthweight-Discordant Study (hereon called LifeSpan). The LSADT, initiated in 1995, was a cohort-sequential study of Danish twins aged 70 years or more, where surviving twins were followed up every second year until 2005 [26]. In 2007, all twin pairs, in which both twins were still alive, were invited to participate. The MADT was initiated in 1998 and included twins randomly selected from birth cohorts spanning 1931–1952. In 2008–2011, a follow-up study was conducted of all eligible twin pairs originally enrolled [25]. The LifeSpan cohort was conducted in 2009–2011 and included twin pairs from the registry, who had a median intrapair difference in birth weight of 0.5 kg [27]. The LifeSpan samples have been analyzed for epigenome-wide association to birth-weight discordance by Tan et al*.* [28], who reported no significant CpGs. The present study population consists of twins for whom both genome-wide epigenetic and genome-wide transcriptomic data were available: 84 twins from the LSADT 2007 wave, 476 twins from the MADT 2008–2010 wave, and 275 twins from the Lifespan cohort. Zygosity of the present study population was determined either by microsatellite markers and/or genome-wide genetic data (LifeSpan and LSADT) or by four questions regarding physical similarity (MADT). The latter has been shown to correctly classify more than 95% of the pairs [29]. Characteristics for the current study population are summarized in Table 1.

**Table 1:** Characteristics of the study population

No. of participants	835
Women (%); Men (%)	407 (48.74%); 428 (51.26%)
Mean age (SD) (range), years	62.78 (15) (30.14–91.7)
Year of blood sample	2007–2011
Zygosity (MZ (%); DZ (%))	785 (94.01%); 50 (5.99%)
No. of cases with cerebrovascular diseases^**#**^ (Prevalent (%); Incident (%)) No. of discordant twin pairs (Prevalent (%); Incident (%))	40 (4.79%); 80 (10.06%) 28 (3.35%); 60 (7.43%)
No. of cases with coronary artery diseases^**¥**^ (Prevalent (%); Incident (%)) No. of discordant twin pairs (Prevalent (%); Incident (%))	86 (10.3%); 76 (10.15%) 41 (4.91%); 44 (5.54%)
No. of cases with arterial and cardiovascular diseases^*^ (Prevalent (%); Incident (%)) No. of discordant twin pairs (Prevalent (%); Incident (%))	110 (13.17%); 165 (22.76%) 41 (4.91%); 70 (8.82%)
No. of cases with diseases of the veins and lymphatic system^**§**^ (Prevalent (%); Incident (%)) No. of discordant twin pairs (Prevalent (%); Incident (%))	110 (13.17%); 54 (7.45%) 56 (6.71%); 39 (5.01%)
Smoking status (current (%), former (%), and never smoker (%))	183 (21.92%); 280 (33.53%); 371 (44.43%)
Mean NonHDL (SD) (range), mmol/L	3.6 (1) (1.59–6.99)
Mean systolic blood pressure (SD) (range), mmHg	148.33 (23) (90–237)
Mean height (SD) (range), cm	168.77 (10) (140–205.4)
Mean weight, (SD) (range), kg	74.85 (15) (35–145)
No. of diabetes cases (%)	30 (3.59%)
No. of hypertension cases^£^ (%)	256 (30.66%)
Statin use (C10AA) (%)	147 (17.6%)

No.: number of, SD: standard deviation, MZ: monozygotic, DZ: dizygotic

^#^Cerebrovascular diseases**:** ICD-8: 430–438 + ICD-10: I60–I69, ¥: Coronary artery diseases: ICD-8: 410–414 + ICD-10: I20–I25, * Arterial and other cardiovascular diseases: ICD-8: 420–429,440–448 + ICD-10: I27–I28, I30–I52, I70–I79, §: Diseases of the veins and lymphatic system: ICD-8: 450–457 + ICD–10: I26, I80, I89, NonHDL: non-high-density lipid-protein level (total cholesterol—high-density lipoprotein), mmol/L: millimolar per liter, mmHg: millimeter of mercury, cm: centimeters, kg: kilo grams. £: hypertension: ICD-8: 400–404 + ICD-10: I10–I15

For all cohorts, data were collected through comprehensive interview-based questionnaries and examinations, as well as blood sampling. Moreover, data on disease diagnoses and survival status were available through linkage to nationwide health registries (see below). Informed consent to take part in the cohorts was obtained from all participants, and the survey was approved by the Regional Scientific Ethical Committees for Southern Denmark (S-VF-19980072, S-VF-20040241, and S-20090033) and conducted in accordance with the Helsinki II declaration.

### Cardiovascular disease diagnoses from the Danish National Patient Registry

The four disease groups analyzed in the present study are listed in Table 1.

International Classification of Diseases (ICD) codes were extracted from the DNPR, which contains all hospital discharges and outpatient visits from all Danish hospitals since 1977 [24]. In the present study, diagnoses were available until the 23rd of November 2022. All national health registries in Denmark are linked via a unique personal identification number assigned to all Danish residents [30]. DNPR holds ICD-8 and ICD-10 codes, with ICD-10 since 1994, while ICD-9 codes were never implemented in Danish registries. Here, ICD codes for all Diseases of the Circulatory Organs (i.e., ICD-10 I00–99 [31]) were defined in overall disease groups according to The Danish Health Data Authority browser [32], the Statistics Denmark, holding the historical information on disease grouping [33], as well as the definitions by the Danish Cause of Death Registry [34]. Consequently, eight groups were initially defined:

1. Rheumatic fever and chronic rheumatic heart diseases (ICD-8: 390–398 and ICD-10: I00–I09),
2. Hypertension (ICD-8: 400–404 and ICD-10: I10–I15),
3. Ischemic heart diseases (ICD-8: 410–414 and ICD-10: I20–I25),
4. Vascular diseases of the brain (ICD-8: 430–438 and ICD-10: I60–I69),
5. Diseases of arteries, arterioles, and capillaries (ICD-8: 440–448 and ICD-10: I28, I70–I79),
6. Other heart diseases (ICD-8: 420–429 and ICD-10: I27, I30–I52),
7. Diseases of the veins and lymphatic system (ICD-8: 450–457 and ICD-10: I26, I80–I89).
8. Other and unspecific diseases of the circulatory system (ICD-8: 458 and ICD-10: 95–99).

As blood pressure was to be included as a covariate in the present study (see below, cf. [35]), group No. 2 was not considered further. For each of the remaining groups, the number of individuals with at least one diagnosis within the disease group in question was counted, including both main and secondary diagnoses. No cases were found for group No.1, and for group No. 8, only diagnoses of low blood pressure (*N* = 16) or unspecific/post-operation diagnoses were found (*N* = 7). Hence, only the five remaining groups were considered in the analysis. Due to a limited sample size, group No. 5 (*N* = 84) was combined with group No. 6 (*N* = 234), similarly to [36]. This group is hereafter referred to as: Arterial and other cardiovascular diseases. Based on the definitions of the WHO [31], group No. 7 was kept as a separate group. Consequently, four disease groups were analyzed in the present study:

- Vascular diseases of the brain, hereon called cerebrovascular diseases (CD),
- Ischemic heart diseases, also known as coronary artery diseases (CADs),
- Arterial and other cardiovascular diseases (AOCDs),
- Diseases of the veins and lymphatic system (DVL).

For each of these four disease groups, the first occurring diagnose date was defined for each individual, and they were then divided into either prevalent or incident cases, i.e., whether the first occurring diagnosis date was before (prevalent) or after (incident) blood sample collection. The reason for this division of cases was to condition on the temporality between expose and outcome; CVD event occurring before blood sample can potentially be reflected in a blood sampling taken after a disease event, whereas biological variation identified in an analysis of CVD events occurring after blood sampling for individuals with no diagnosis prior to the blood sampling is in principle predictive of future CVD events. In the subsequent data analyses (see below) of each of the disease groups, analysis of prevalent cases was performed by comparing cases to the rest of the study population (i.e., the individuals with no diagnose before blood sampling within the disease group in question). Incident cases were analyzed based on time to first diagnosis (incident cases) or time to death or end-of-follow-up (individuals remaining diagnose free) and excluding the prevalent cases from the disease group in question. For analysis of incident cases, information on survival status was retrieved from the Danish Central Person Register, which is continuously updated [37]. Finally, within each of the four disease groups, the number of discordant twin pairs was identified. The prevalent discordant cases were twin pairs, in which one twin was diagnosed with the disease before blood sampling, while the co-twin was not. The incident discordant cases were twin pairs, in which one twin obtained a diagnosis after blood sampling, while the co-twin remained disease-free or died, as well as pairs, in which both twins obtain a diagnosis, although at different time points after blood sampling.

### Genome-wide omics data obtained for DNA and RNA from the same individuals

Detailed descriptions of the laboratory methods and quality control of the Infinium HumanMethylation450K BeadChip (Illumina, San Diego, CA, USA) methylation data, and of the Agilent SurePrint G3 Human GE 8 × 60K Microarray (Agilent Technologies) transcriptomic data can be found in [38] and [39], respectively. A brief overview can be found in the Supplementary Methods. Annotation of the DNA methylation data was done using GRCh37/hg19 (as per year 2018), applying the annotation file from [40], as recommended by Illumina Inc. (Illumina, San Diego, CA, United States) (personal communication), while the transcriptomic data were annotated using GRCh37/hg19, applying the annotation file supplied by Agilent (as per November 2022). This annotation among others includes information of names of genes located in the specific genomic position of the CpG or probe, these gene names were used for the subsequent bioinformatics analyses (see below).

Lastly, for both genome-wide DNA methylation data and genome-wide gene expression data, the biological material from each of the three cohorts had been analyzed on different occasions, hence, each dataset had been processed individually before the datasets were merged in the present study (see Supplementary Methods for details). Only reoccurring CpGs and gene expression probes across the three cohorts were used for the present study, resulting in 451,547 CpGs and 33,869 probes for statistical analyses. To achieve a distribution more fit for statistical testing, the β-values of the DNA methylation data were transformed to M-values by quantile transformation [41].

### Survey phenotype data considered as covariates

Based on the HeartScore algorithm, developed by the European Association of Preventive Cardiology [35], for estimation of the risk of CVD disease events, the following covariates were considered: age, sex, self-reported smoking status (never, former, and current smoker), systolic blood pressure (mmHg), and non-high-density lipoprotein cholesterol (nonHDL) level (mmol/L). The latter is defined as the level of high-density lipoprotein cholesterol (HDL) subtracted from the total cholesterol level in blood. Standard systolic blood pressure measurements of the upper right arm, and blood lipid levels, by routine hospital measurements, were available for 740 (89%) and 357 (43%), respectively, of the 835 individuals in the study population. In order to consider these two variables as covariates, imputation was performed using the Multiple Imputation by Chain Equations (MICE) method. Moreover, sensitivity analyses of real versus imputed values, and sensitivity analyses with or without inclusion of the two variables were performed and led to the same overall conclusions (see Supplementary Methods for details).

Moreover, blood cell counts were also considered, as blood cell composition can bias molecular epidemiology studies performed in blood samples. Data were available as hematological counts of leukocytes subtypes (neutrophils, monocytes, basophils, eosinophils, and lymphocytes) for 763 (91%) out of the 835 individuals. Cell counts for the remaining individuals had previously been imputed using epigenome-wide data with a modified version of the PredictCellComposition method (see [42] for details).

Before statistical analyses, an exploratory principal component analysis (PCA) was initially performed (similarly to [38]) of all survey and register data considered in the present study as well as of the DNA methylation data and the gene expression data, respectively, with the aim to examine the correlation between potential confounder variables and the principal components (PCs) of the omics data. Subsequently, either the confounder variable or the PC was included statistical analyses (see Supplementary Methods for details).

For descriptive purposes, information on height, weight, overall diabetes status, and medication use was included. Height and weight were measured for the LifeSpan and MADT individuals, whereas they were self-reported for LSADT individuals. With respect to medication, the use of statins (ATC code C10AA) was based on self-report and was defined following the ATC classification guideline [43].

### Statistical analyses

All statistical analyses were performed in R version 4.1.0, while initial data handling and cleaning of survey and register data were conducted using STATA 17.0.

For all statistical models (see below), imputation of systolic blood pressure and nonHDL was performed using MICE by creating 20 datasets, 20 iterations for each dataset and pooling the results applying Rubin’s rules (see Supplementary Methods for details). The R libraries including tidyverse, ggpubr, parallel, mice, and broom.mixed were used. Adjusting for multiple testing was performed using Benjamini–Hochberg FDR correction method [44]. Lastly, volcano plots were constructed using the ggplot2 library to visualize patterns in the direction of estimates.

#### Analyses of prevalent cases

EWAS and TWAS were performed using linear regression models in the lme4 library with the single DNA methylation (CpG) level and the gene expression level, respectively, as the outcome and the given disease group status as the explanatory variable. EWAS and TWAS were conducted both at the individual-level and at the twin pair-level.

The individual-level analysis was performed applying a linear mixed effect regression model with twin pair ID as a random factor in order to take the within-pairs’ dependency into account. The following covariates were included for both EWAS and TWAS: age at blood sampling, sex, nonHDL, systolic blood pressure, and smoking status as fixed effects, and an omics dataset variable as an additional random effect. Moreover, PC3 and PC2 were included for EWAS and TWAS, respectively, and reflected the cell counts (see Supplementary Methods).

In the twin pair-level analysis, intrapair differences in twin pairs discordant for disease status were investigated. The intrapair difference was calculated for a given variable by subtracting the variable value of the co-twin without a diagnosis from that of the co-twin with a diagnosis. A linear regression model was applied with the intrapair difference at DNA methylation level and the gene expression level, respectively, as the outcomes, and with the intrapair differences in the following covariates as the exposures: nonHDL, systolic blood pressure, smoker status, and PC2 in TWAS or PC3 in EWAS. A dataset variable was not included in the intrapair analyses, as the biological samples from the same twin pair had been analyzed on the same array and within the same cohort.

#### Analyses of incident cases

For analysis of incident disease cases, Cox proportional hazards regression using the Survival library was conducted, both at the individual-level and at the twin pair-level.

The individual-level analysis was performed using age as the underlying timescale (delayed entry at blood collection) and adjusted for sex, nonHDL, systolic blood pressure, smoking status, and PC2 in TWAS or PC3 in EWAS. We used age as the underlying time scale to ensure proper age adjustment and to allow a nonlinear relation between the molecular marker analyzed and the risk of CVD. Within pair dependence was adjusted in the models to account for dependency between twins in each twin pair. Individuals who died during follow-up without obtaining a diagnosis in the disease group in question were censored at time of death.

The twin pair-level analysis was performed using the stratified Cox regression, where the baseline hazard functions was pair specific.

### Bioinformatic analyses of overlapping genes

The CpG sites investigated in EWAS, and the probes investigated in TWAS were annotated. This annotation includes information of the names of genes located in the specific genomic position of the CpG or probe. These gene names were used in gene set enrichment analysis (GSEA) with the aim to explore the biological pathways in which the identified CpGs and probes take part. Specifically, for each analysis, i.e., within each of the four disease groups and within each of the four statistical models (individual-level analysis and twin pair-level analysis of prevalent cases as well as individual-level analysis and twin pair-level analysis of incident cases), the overlap in gene names of the CpGs (EWAS) with FDR < 0.05 and the gene names of the probes (TWAS) with FDR < 0.05 was explored. Similarly, overlapping genes were inspected using a cutoff *p* < 0.05. As some CpGs or probes might be annotated to different genes or different aliases, and as different CpGs and probes might be annotated to the same gene, the lists of genes used to find the overlaps included all unique genes identified for a given analysis. Subsequently, for these 16 lists of EWAS-TWAS overlapping gene names (i.e., 4 disease groups times 4 statistical models) the overlap in EWAS-TWAS overlapping gene names from the individual-level analysis, and EWAS-TWAS overlapping gene names from the twin pair analysis were found for each disease group and within prevalent and incident cases. Such an overlap holds the EWAS-TWAS overlapping genes identified in the individual-level analysis and confirmed in the twin pair-level analysis, i.e., the genes confirmed when reducing the potential confounding due to shared genetic factors and early life environment. Such genes must be considered the most relevant genes when exploring the association of molecular markers to CVD. These 8 lists of confirmed genes (i.e., 4 disease groups times prevalent or incident cases) were used for GSEA in the GSEA database [45–47] with application of the Kyoto Encyclopedia of Genes and Genomes (KEGG) and Reactome databases. Lastly, the overlap in genes between analyses of prevalent and analyses of incident cases, as well as all cases, was also explored. The lists of genes identified and used in GSEA were below the maximum number of 500 genes, which the GSEA database can handle with certainty [48].

Finally, interaction networks were also identified for the 8 list of genes by application of StringApp in Cytoscape [49]. In the displayed networks, genes not connected to other genes were not visualized, and for complex networks only the most connected genes are visualized. Unconstrained networks can be found in the Supplementary Results.

## Results

The study design of the present study is depicted in Fig. 1. In the present study, we explore the association between variation in DNA methylation and gene expression levels and diagnoses of the circulatory system in 835 twins. We perform association analyses both at the individual-level and at the twin pair-level, the latter reducing the potential confounding introduced by genetic variation and variation in early life environment. Descriptive characteristics of the study participants can be seen in Table 1. As seen from this table, between 4.8% and 13.2% of the study population had a cardiovascular disease event before blood sampling (prevalent cases), depending on the disease group. Of the individuals without disease diagnose at the time of blood sampling, between 7.5 and 22.8% experienced a cardiovascular disease event after blood sampling (incident cases), depending on the disease group.

### Epigenome-wide association studies and transcriptome-wide association studies of cardiovascular disease groups

TWAS and EWAS were performed for four different groups of CVD, i.e., cerebrovascular diseases (CD), coronary artery disease (CAD), arterial and other cardiovascular diseases (AOCD), and diseases of the veins and lymphatic system (DVL) in the entire twin population. For each disease group, analyses of both prevalent and incident cases were performed, as well as analyses of individual and intrapair differences. In TWAS, 33,869 transcripts were studied, and, in EWAS, 451,547 CpG sites were investigated.

The results of the association analyses holding a p value below 0.05 are listed in Supplementary Results, part 1. The number of CpGs with a p value below 0.05 in the EWAS ranged from 10,690 to 53,417 (corresponding to 2.4–11.8% of all CpGs) depending on the disease group and statistical analysis, while for TWAS, the range was 741–5273 probes (corresponding to 2.2–15.6% of all probes) depending on the disease group and statistical analysis.

Correction for multiple testing (FDR < 0.05) revealed 3 CpGs and 1 probe in the individual-level analysis of prevalent cases (see Table 2): cg16541931, cg04329510, and cg09378783 were all found associated with CD and annotated to the promoter region of *GPR158* (G protein-coupled receptor), the 3′UTR of *C5AR1* (complement C5a receptor 1) and to the gene body of *EFGR* (epidermal growth factor receptor), respectively, while A_19_P00806441, associated to DVL, was annotated to the *LOC105370365* RNA gene. Moreover, two CpGs displayed association in the individual-level analysis of incident CAD: cg06567227 and cg06714480 annotated to the gene body of *SIM1* (SIM BHLH transcription factor 1) and to the 5′UTR of *NEUROD1* (neuronal differentiation 1) and *CERK* (Ceramide Kinase), respectively. Finally, none of the CpGs or probes found to have a p value below 0.05 in the twin pair-level analyses passed correction for multiple testing.

**Table 2:** Significant CpG sites and probes associated with a disease group (FDR < 0.05) in the individual-level analyses

Analysis	Probe/CpG	Coef	SE	*P* value	FDR	Gene
EWAS of prevalent cerebrovascular diseases	cg16541931	0.14	0.03	3.48E−8	0.0079	GPR158;GPR158-AS1
	cg09378783	− 0.26	0.05	2.32E−8	0.0079	EGFR
	cg04329510	− 0.17	0.03	1.58E−7	0.0238	C5AR1
EWAS of incident coronary artery diseases	cg06567227	− 1.28	0.21	6.64E−8	0.03	SIM1
	cg06714480	2.05	0.35	1.33E−7	0.03	CERKL;NEUROD1
TWAS of prevalent diseases of the veins and lymphatic system	A_19_P00806441	− 0.07	0.01	9.32E−8	0.0032	LOC105370365

Coef.; coefficient, SE; standard error, FDR: false discovery rate, EWAS: epigenome-wide association study, TWAS: transcriptome-wide association study

### Genes in overlap between EWAS and TWAS

As seen in the previous section, no genes held both a CpG and a gene expression probe with an FDR corrected p value below 0.05. Hence, the 10,690–53,417 CpGs found to have a p value below 0.05 in the EWAS, and, in the TWAS, the 741–5,273 probes found to have a p value below 0.05 were investigated. They were annotated to 8634–21,561 unique genes from EWAS, and to 621–4,332 unique genes from TWAS, respectively. These unique genes were used to inspect the overlap in genes between EWAS and TWAS analyses within each disease group and within prevalent and incident cases, with the purpose to investigate the biology of these overlapping genes in GSEA (see below). Firstly, 8 gene lists of EWAS-TWAS overlapping genes were found for the prevalent cases (i.e., 4 disease groups times 2 analyses (individual-level and twin pair-level analyses)). Similarly, 8 gene lists of EWAS-TWAS overlapping genes were found for analysis of incident cases (i.e., 4 disease groups times 2 analyses (individual-level and twin pair-level analyses)). These 16 lists of EWAS-TWAS overlapping genes are listed in Supplementary Results, part 2. The number of overlapping genes ranged between 217 and 2007 depending on disease group and analysis. As the present study population was twins, we further identified the overlap between the EWAS-TWAS overlapping genes of the individual-level analysis and the EWAS-TWAS overlapping genes of the twin pair-level analysis within each disease group and within prevalent and incident cases. Such genes are the genes identified in the individual-level analysis, yet also confirmed in the twin pair analysis, the latter effectively reducing the potential confounding introduced by genetic variation and variation in early life environment. As such, these genes can be considered the most interesting candidates when investigating the association between variation in DNA methylation and gene expression levels and a given phenotype of interest. These 8 lists of genes (4 disease groups in prevalent analysis, and 4 disease groups in incident analysis) are listed in Supplementary Results, part 2. For prevalent cases, 136, 447, 391, and 109 genes were found for the CD, CAD, AOCD, and DVL, respectively, whereas for the incident cases 34, 104, 306, and 42 genes were found for the CD, CAD, AOCD, and DVL, respectively. Subsequently, volcano plots with all CpGs or probes displaying a p value below 0.05 were constructed (see Supplementary Figures) with the aim to explore the direction of the effect of the CpGs or probes located in the overlapping genes. As seen from these volcano plots, no general pattern in the direction of effect was seen. Subsequently, the overlapping genes were investigated in GSEA and displayed in gene networks.

#### Gene network and gene set enrichment analyses of the genes identified for diagnosis after blood sampling

With the aim to identify biological variation associated with future CVD, the incident CVD cases were analyzed first. Such analysis potentially identifies predictive biomarkers of CVD. For the incident cases, the number of genes confirmed in the twin pair-level analysis was 34, 104, 306, and 42 for the CD, CAD, AOCD, and the diseases of DVL, respectively (see Supplementary Results, part 2).

**Fig. 2:**
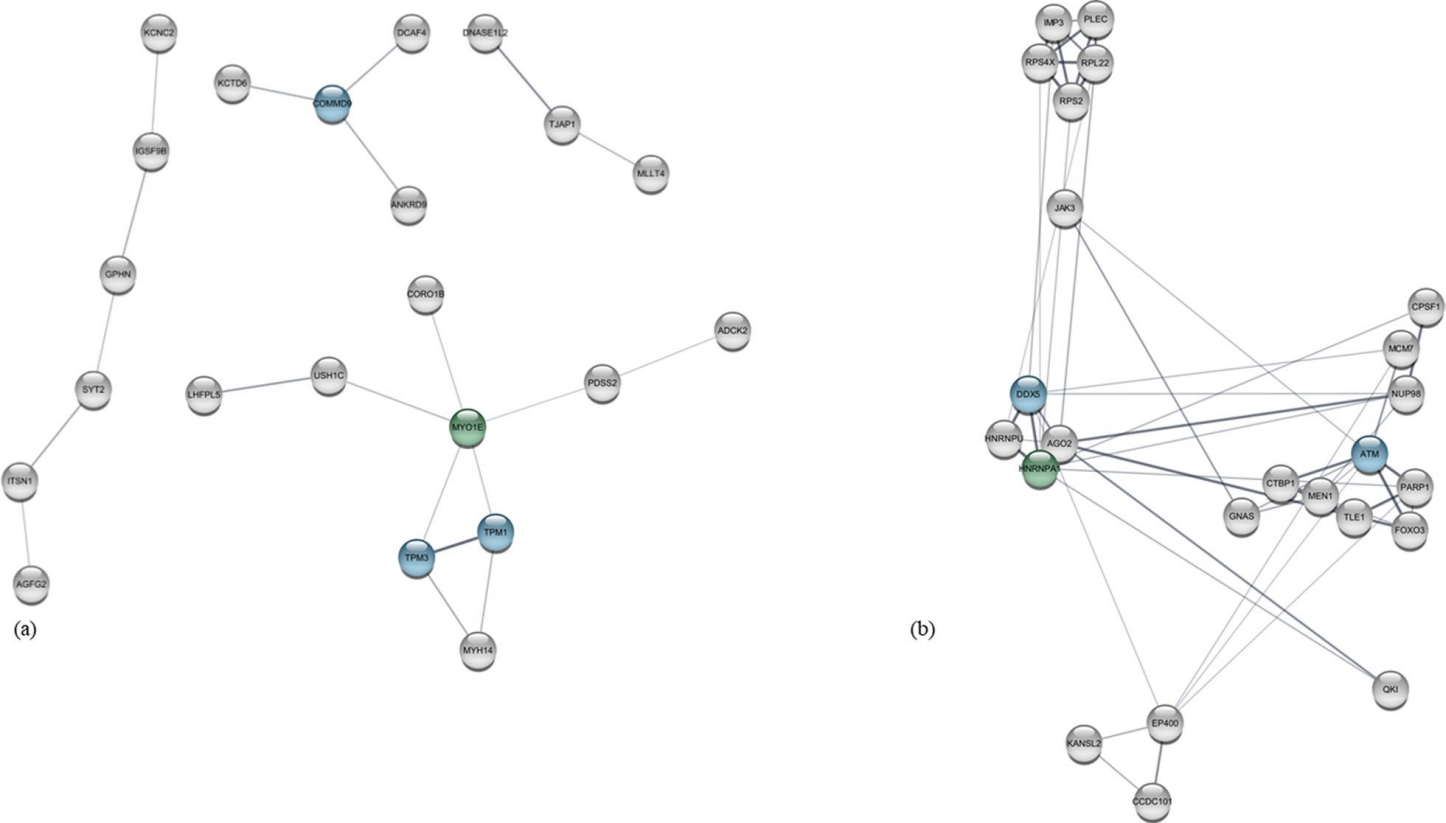
Interaction networks for incident cases of coronary artery diseases and arterial and other cardiovascular diseases. Interaction networks for incident cases of (**a**): coronary artery disease and (**a**) arterial and other cardiovascular diseases. Genes colored green represent the most connected genes, whereas blue represents the second-most connected gene. For (**b**) only genes displaying minimum 5 connections are displayed

Interaction networks for each of these four groups of genes are displayed in Supplementary Results, part 3. CD displayed no networks, whereas DVL showed networks with maximum two interactions. Figure 2 display the interaction networks for CAD and AOCD: as seen from the figure, the most interacting gene for CAD was *MYO1E* (myosin 1E), *COMMD9* (the COMM Domain Containing 9), *TPM1* (Tropomyosin 1), and *TPM3* (Tropomyosin 3), whereas for AOCD, the most connected gene was *HNRNPA1* (Heterogeneous Nuclear Ribonucleoprotein A1) followed by *ATM* (Ataxia-telangiectasia mutated), and *DDX5* (DEAD-Box Helicase 5).

When performing GSEA on these gene lists, we found no pathways for CD, or CAD, whereas 4 pathways were found for the AOCD, and 2 pathways were observed for DVL (see Table 3). As seen from the table, the pathways reflected cell–cell communication, and metabolisms of proteins and RNA, gene expression, and chromatin organization.

**Table 3:** Pathways identified for incident CVD groups

CVD group	Hierarchical group	Gene set name	# Genes in Gene Set (K)	# Genes in Overlap (k)	k/K	*P* value	FDR q value
Arterial and other cardiovascular diseases	Metabolism of proteins	Posttranslational protein modification (R-HSA-597592)	1442	30	0.0208	2.46E−7	4.52E−4
	Gene expression (Transcription)	RNA Polymerase II Transcription (R-HSA-73857)	1393	28	0.0201	1.23E−6	1.14E−3
	Metabolism of RNA	Metabolism of RNA (R-HSA-8953854)	714	17	0.0238	2.03E−5	1.25E−2
	Chromatin organization	Chromatin modifying enzymes (R-HSA-3247509)	274	10	0.0365	3.39E−5	1.56E−2
Diseases of the veins and lymphatic system	Cell–Cell communication	Tight junction interactions (R-HSA-420029)	30	3	0.1000	3.58E−6	6.58E−3
	Cell–Cell communication	Cell–cell junction organization (R-HSA-421270)	65	3	0.0462	3.76E−5	3.46E−2

Arterial and other cardiovascular diseases (ICD-8: 420–429,440–448 + ICD-10: I27–I28, I30–I52, I70–I79), diseases of the veins and lymphatic system (ICD-8: 450–457 + ICD-10: I26, I80, I89), FDR: false discovery rate

#### Gene network and gene set enrichment analyses of the genes identified for diagnosis before blood sampling

Subsequently, the prevalent cases were analyzed. Genes identified in such analyses associate to CVD events occurring before blood sampling and, hence, potentially reflect physiological processes occurring as a consequence of disease. For the prevalent cases, the number of genes confirmed in the twin pair-level analysis were 136, 447, 391, and 109 for the CD, CAD, AOCD, and DVL, respectively (see Supplementary Results, part 2).

**Fig. 3:**
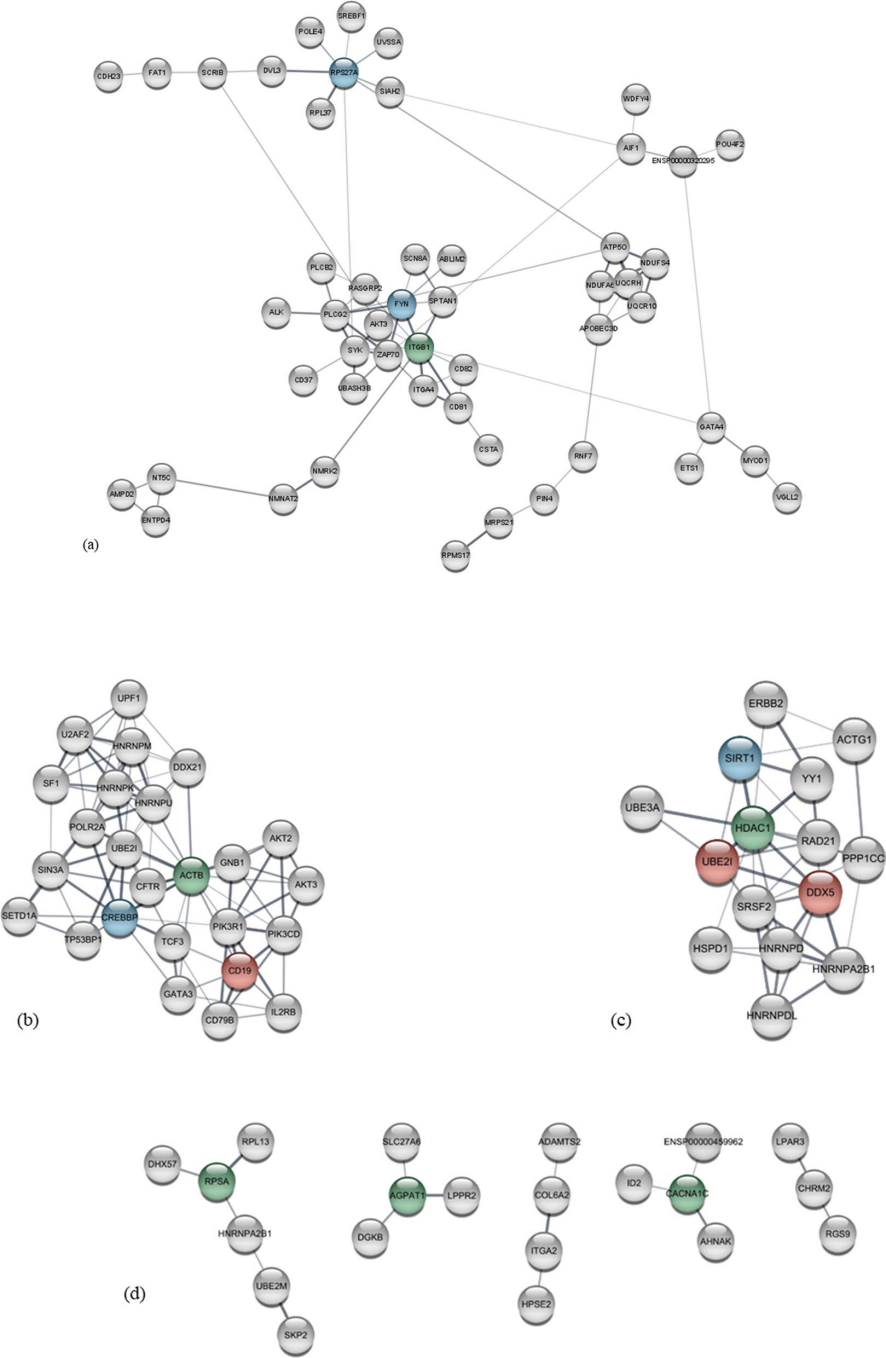
Interaction network for the overlapping genes identified for prevalent CVD cases. Interaction network for the overlapping genes identified for prevalent CVD cases. **a** cerebrovascular diseases, **b** coronary artery disease, **c** arterial and cardiovascular diseases and **d** diseases of the veins and lymphatic system. Genes colored green represent the most connected genes, blue represents the second-most connected gene, and red represents the third-most connected gene. For **b** and **c** only genes displaying minimum 15 connections are displayed, while for (**d**) it is the largest networks

Full interaction networks for each of these four groups of genes can be found in Supplementary Results, part 3, while networks with the genes displaying the most connections are displayed in Fig. 3. As seen in the figure, the CD, CAD, and AOCD displayed rather dense networks, whereas the network for DVL was less dense. The three most connected genes for CD were *ITGB1* (integrin subunit beta 1) and *FYN* (FYN proto-oncogene, Scr family tyrosine kinase) that were located in the same subcluster, and *RPS27A* (the ribosomal protein S27a gene) located in another subcluster. For CAD, the three most connected genes were *ACTB* (Actin Beta), *CREBBP* (CREB Binding Protein), and the *CD19* (CD19 Molecule). For AOCD, the three most connected genes were *HDAC1* (Histone Deacetylase 1), *SIRT1* (Sirtuin 1), *DDX5*, and *UBE2I* (Ubiquitin-Conjugating Enzyme E2 I). For DVL, the most connected genes were the *AGPAT1* (1-Acylglycerol-3-Phosphate O-Acyltransferase 1), *CACNA1C* (Calcium Voltage-Gated Channel Subunit Alpha1 C), and *RPSA* (Ribosomal Protein SA).

When performing GSEA using each of these gene lists 48, 100, and 100, we found pathways with an FDR < 0.05 for CD, CAD, and AOCD, respectively (see Supplementary Results, part 4). Generally, the majority of the identified pathways were involved in signal transduction, immune system, and disease. No pathways were found for the 109 genes found for DVL.

#### Overlap in genes identified in analyses of prevalent CVD and incident CVD within each cardiovascular disease group

Finally, the overlap in genes found for prevalent cases and for the incident cases were inspected per CVD group. For CAD, 6 overlapping genes were found, whereas for AOCD, 19 genes were found (see Supplementary Results part 2, sheet 3). Performing of GSEA of these overlapping genes revealed no significant pathways. Gene network analysis revealed no networks for CAD, while *PIGG* (Phosphatidylinositol Glycan Anchor Biosynthesis Class G) and *UVSSA* (UV-Stimulated Scaffold Protein A) were the only two genes to show connection for AOCD. Lastly, when similarly investigating the overlap in GSEA, we found pathways for prevalent cases and for the incident cases per CVD group, we found four Reactome pathways for AOCD. These were “Posttranslational protein modification (R-HSA-597592),” “RNA polymerase II transcription (R-HSA-73857),” “Metabolism of RNA (R-HSA-8953854),” and “Chromatin modifying enzymes (R-HSA-3247509).”

## Discussion

Globally, cardiovascular diseases (CVDs) remain the leading cause of death [1]. Studies using DNA methylation as well as gene expression data to investigate CVD development are rare. In such studies, a major challenge is to reduce genetic confounding. Hence, application of twin data is ideal to decrease such confounding. Additionally, registry data are likely to be less biased than self-reported data. Thus, we here use twin register data of 835 twins to study the relationship between CVD development and DNA methylation and gene expression.

First, analyses of DNA methylation and gene expression data were performed individually. Three gene regions were found in EWAS of prevalent cerebrovascular diseases (CD) holding for multiple testing (FDR < 0.05): *GPR158/GPR158-AS1*, *C5AR1,* and *EGFR*, encoding a protein likely involved in G protein-coupled receptor activity/its antisense RNA, a receptor of complement 5a, and a growth factor receptor, respectively. *GPR158* is highly expressed in the brain and associates to nervous system-related tumors [50] and cardiac fibrosis [51], whereas *C5AR1* associates to atherosclerosis [52], coronary artery disease (CAD) [53, 54], and CD [55]. *C5AR1* has also been presented as a potential drug target for reducing inflammation [56, 57]. *EGFR* is highly expressed in the neural stem cells and plays a role during embryogenesis and organogenesis, including the heart. In the central nervous system, *EGFR* is, among others, involved in the neural stem cells’ pool maintenance and axonal regeneration. Moreover, the two gene regions found in EWAS of incident CAD (FDR < 0.05), i.e., *SIM1* and *NEUROD1*/*CERKL*, encode transcription factors (*SIM1* and *NEUROD1*) or a protein with ceramide kinase-like domains, yet with unknown function (*CERKL*). Although these genes have not been linked directly to CVD, they are linked to CVD risk factors; *SIM1* has a role in neuronal differentiation of the hypothalamus, which is critical for food consumption regulation [58], and loss-of-function mutations in *SMI1* associate with increased risk of obesity [59]. *NEUROD1* is expressed by pancreatic and nervous tissues and is essential for the development of beta cells and diabetes [60], which have been reported to influence cardiac function, as well as inflammation [61]. Lastly, *LOC105370365* notably found in TWAS of prevalent diseases of the veins and lymphatic system (DVL) encodes a noncoding RNA of unknown function. Overall, although not all of these gene regions have previously been linked directly to the CVD phenotype, for which they display association in the present study, most do relate to relevant biology, CVD traits, or risk factors.

Furthermore, the overlap in genes identified in EWAS and TWAS was found, and, subsequently, the genes found both in the individual-level analysis and in the twin pair-level analysis were identified. The fact that these genes are confirmed, when the potential confounding effects of genetics and early life environment were reduced, makes them the most noteworthy genes for identifying nongenetic markers to CVD. These overlap genes were inspected by pathway and network analyses. Interestingly, for the many pathways found for the prevalent CVD events, the most frequent hierarchical groups were related to signal transduction, the immune system, diseases, metabolism and metabolism of RNA and proteins. While the fewer pathways found for the incident CVD events, the hierarchical groups were related to cell–cell communication, metabolisms of proteins and RNA, gene expression, and chromatin organization. This could potentially reflect an overall physiological response to disease in the prevalent cases, compared to a more regulatory effect for the incident cases. In any case, this difference indicates that investigation of prevalent cases and incident cases indeed reflect different biology, and it calls for longitudinal studies of incident cases to identify biomarkers with true predictive abilities. Finally, when constructing networks with the overlapping genes, more dense networks were in general found for prevalent cases than for incident cases, potentially reflecting differences in biology. For prevalent cases, the most connected genes in these dense networks were: *ITGB1* for CD, *ACTB* for CAD, and *HDAC1* for AOCD, encoding an integrin subunit, actin beta, and a histone deacetylase, respectively. Integrins are membrane receptors involved in cell adhesion and recognition in a variety of processes including hemostasis, tissue repair, and immune response, where studies have shown that *ITGB1* has a protective role in CVD development [62–64]. Actins are highly conserved proteins involved in cell motility, structure, integrity, and intercellular signaling, where *ACTB* is found to be associated with CAD [65] and CD [66]. *HDAC1* is involved in deacetylation of lysine residues on the N-terminal part of the core histones, giving a tag for epigenetic repression, and related to transcriptional regulation, as well as cell cycle progression. *HDAC1* is a negative regulator of cardiomyocytes that is found to be associated with CVD development [67] and is suggested as a potential drug target [68]. For the incident analyses, the networks were less dense; for CAD, the largest network was centered around *MYO1E*, which encodes a non-muscle membrane-associated class 1 myosin, expressed in the kidneys, where it helps the capillaries resist hydrostatic pressure at the glomerular filtration barrier by generating tension [69]. *MYO1E* mutations are associated with focal segmental glomerulosclerosis, characterized by an increased risk of CVD, primary due to increase in dyslipidemia and hypertension [70]. In the network *MYO1E* was among others connected to *TPM1* and *TPM3*, which encode Tropomyosin 1 and 3, respectively. Tropomyosin 1 plays a role in calcium mediated muscle contraction and associates to several heart diseases, including hypertrophic cardiomyopathy and CAD [71, 72]. Mutations in *TPM3* can result in congenital muscle weakness due to reduced calcium-sensitivity [73], and potentially to an increased risk of CVD [74]. Another cluster for CAD held the *COMMD9* as the most central gene. *COMMD9* is expressed in the kidneys and is involved in sodium ion transport and cholesterol homeostasis [75]. Changes in sodium transportation can result in blood pressure alterations, which can lead to CVD [76]. Interestingly, the Na^+^–Ca^2+^ exchanger is a vital regulator of Ca^2+^ homeostasis in cardiomyocytes and thereby an essential modulator of cardiac contractile function [78]. The network for incident AOCD had *HNRNPA1* as the most connected gene. *HNRNPA1* encodes a mRNA splicing factor affecting gene expression, e.g., *HNRNPA1* reduces expression of 3-hydroxy-3-methylglutaryl-Coenzyme A reductase (*HMGCR*), which encodes a rate-limiting enzyme of the cholesterol biosynthesis pathway. Reduced expression of *HMGCR* leads to reduced cholesterol synthesis and increased low-density lipoprotein (LDL) and apolipoprotein B (ApoB) uptake from the blood. Hence, *HNRNPA1* has been suggested to have cholesterol lowering effects similar to statins [77]. *HNRNPA1* is a regulator of vascular smooth muscle cell function and neointimal hyperplasia, i.e., the proliferation and migration of vascular smooth muscle cells in the innermost layer of the arteries, which results in thickening of the arterial wall and atherosclerosis [79]. Central for the same network were also *ATM* and *DDX5*, which encodes a DNA repair protein and an RNA helicase, respectively. *ATM* deficient cells are characterized by hyperlipidemia, which promotes atherosclerosis and CVD [80, 81], and several studies have associated *ATM* to CVD [82, 83]. *DDX5* is expressed in the smooth muscle cells in arteries, among others, and is suggested to have a protective role in neointimal hyperplasia [84]. Consequently, *DDX5* has been suggested as a potential target for vascular therapies [85]. Lastly, despite the fact that different genes, pathways, and networks were found in the analyses of either prevalent or incident cases, some genes were found in both. Among genes found in both analyses were *MYO1E* (for incident CAD) and *DDX5* (for incident AOCD). Such genes might be considered generalist genes, as the DNA methylation and gene transcription levels change as a consequence both of the disease (prevalent cases) as well as prior disease (incident cases).

The strength of the present study is primarily the use of register data from a nationwide registry that makes it possible to investigate all CVD diagnoses, which are less biased than self-report, and, furthermore, enables the investigation of both prevalent and incident cases. Another great strength is the use of twin data, which enabled us to examine intrapair differences, facilitating a detailed study of the gene expression and DNA methylation differences associated with CVD, while at the same time controlling for genetics and shared early life environmental confounders. Lastly, epigenetic data and gene transcription data are available from the same individuals in the present study, which is indeed rare in this line of research. To investigate both types of data, it is beneficial to investigate biological mechanisms due to the integration of different omics layers providing a more biologically holistic view compared with single omics studies. However, the study also has a number of limitations; first of all, the use of blood samples means that the molecular markers measured reflect the overall biology of the individuals, and not the biology specific to for instance the heart muscle cells or brain cells. On the other hand, identification of molecular markers applicable in a blood sample would have great potential due to the non-invasive nature of the sampling. Moreover, we conducted an explorative study, as genes with a *p* value below 0.05, yet not holding for multiple testing, were investigated. Hence, we cannot exclude false positive findings among these genes, and replication studies are needed to verify the present findings. Nevertheless, considering previous estimations of the statistical power of the discordant twin pair design relative to the classical case control study [22], the power of the twin pair analyses of the present study including 28–70 twin pairs appears to be reasonably good (considering a power > 80, a *p* value threshold of 10^–6^, and a heritability of 60%) [22]. The individual-level analysis (including 40–165 cases) is, on the other hand mostly likely under powered for the most part. In any case, as the findings of the present study are conditioned on the findings of the twin pair analyses, this is less of an issue and highlights the strength of including twin pairs in molecular studies as compared to including singletons only. Lastly, the incident CVD cases in the present study were defined as not having an CVD diagnosis within the CVD group of interest before blood sampling. However, we cannot exclude that pathological changes were already present at blood sampling, as e.g., CAD develops over years. So, not even conditioning on absence of diagnosis before blood sampling can ensure that the biology measured does not, to some degree, reflect initial disease.

In conclusion, we here present a study of diagnoses in the entire group of Diseases of the Circulatory Organs obtained from a nationwide registry in 835 twins for which both genome-wide epigenetic and genome-wide gene transcription data were available. Investigation of genes found in both layers of biology, as well as in the analysis of both individuals and twin pairs points to novel candidate genes and pathways. These markers could potentially be beneficial in the detection of both existing and future CVD. However, verification in additional study populations is needed.

### Supplementary Information

Additional file 1. Supplementary Methods.

Additional file 2. Supplementary Results, part 1.

Additional file 3. Supplementary Results, part 2.

Additional file 4. Supplementary Results, part 3.

Additional file 5. Supplementary Results, part 4.

Additional file 6. Supplementary Figures.

## Data Availability

No datasets were generated or analyzed during the current study.

## Abbreviations

ApoB: Apolipoprotein B
AOCD: Arterial and other cardiovascular diseases
BMI: Body mass index
CAD: Coronary artery disease
CD: Cerebrovascular diseases
CVD: Cardiovascular diseases
DNPR: Danish National Patient Registry
DVL: Diseases of the veins and lymphatic system
EWAS: Epigenome-wide association studies
GSEA: Gene set enrichment analysis
GWAS: Genome-wide association studies
HDL: High-density lipoprotein cholesterol
ICD: International Classification of Diseases
KEGG: Kyoto Encyclopedia of Genes and Genomes
LDL: Low-density lipoprotein
LSADT: The Longitudinal Study of Aging Danish Twins
MADT: The Middle Age Danish Twin study
MICE: Multiple Imputation by Chain Equations
NonHDL: Non-high-density lipoprotein cholesterol
PCA: Principal component analysis
TWAS: Transcriptomic-wide association studies

## Genes

*ACTB*: Actin Beta
*AGPAT1*: 1-Acylglycerol-3-Phosphate O-Acyltransferase 1
*ATM*: Ataxia-telangiectasia mutated
*C5AR1*: Complement C5a receptor 1
*CACNA1C*: Calcium Voltage-Gated Channel Subunit Alpha1 C
*CERK*: Ceramide Kinase
*COMMD9*: COMM Domain Containing 9
*CREBBP*: CREB Binding Protein
*DDX5*: DEAD-Box Helicase 5
*EFGR*: Epidermal growth factor receptor
*FYN*: FYN proto-oncogene, Scr family tyrosine kinase
*GPR158*: G protein-coupled receptor
*HDAC1*: Histone Deacetylase 1
*HMGCR*: 3-Hydroxy-3-methylglutaryl-Coenzyme A reductase
*HNRNPA1*: Heterogeneous Nuclear Ribonucleoprotein A1
*ITGB1*: Integrin subunit beta 1
*MYO1E*: Myosin 1E
*NEUROD1*: Neuronal differentiation 1
*PIGG*: Phosphatidylinositol Glycan Anchor Biosynthesis Class G
*RPS27A*: Ribosomal protein S27a
*RPSA*: Ribosomal Protein SA
*SIM1*: SIM BHLH transcription factor 1
*SIRT1*: Sirtuin 1
*TPM1*: Tropomyosin 1
*TPM3*: Tropomyosin 3
*UBE2I*: Ubiquitin-Conjugating Enzyme E2 I
*UVSSA*: UV-Stimulated Scaffold Protein A
