# Supplementary material for: Multi-omics association study of DNA methylation and gene expression levels and diagnoses of cardiovascular diseases in Danish Twins
Source: Clin Epigenetics. 2024 Aug 26;16:117. doi: 10.1186/s13148-024-01727-6 (PMC11348607; doi:10.1186/s13148-024-01727-6)
Supplement: Supplementary file 1 — Additional file 1. Supplementary Methods. [file 13148_2024_1727_MOESM1_ESM.docx]

**Supplementary Information**

1. **Genome-wide omics data**

The current study applied two kinds of genome-wide omics data: epigenomics in form of DNA methylation data, as well as transcriptomics in form of gene expression data. The genome-wide DNA methylation data was drawn from three datasets (one for each cohort) obtained at three different occasions together holding 952 samples (1), while the genome-wide gene expression data was extracted from three datasets (one for each cohort) obtained at three different occasions holding altogether 935 samples (2). The overlap in individuals between the genome-wide DNA methylation data and the genome-wide gene expression data was 835 individuals, which were investigated in the present study. DNA methylation, respectively RNA, from the co-twins within a given twin pair were always analyzed on the same array in order to avoid technical variation when investigating intra-pair differences. Hence, as the findings of the present study is conditioned on the twin pair analysis, it must be considered less prone to batch effects as compared to investigation of singletons. Nevertheless, as the DNA methylation datasets, respectively the gene expression datasets, were generated at different occasions, a dataset variable describing each dataset, or a principal component (see below), were included in the statistical analyses in the present study in order to account for the potential between dataset variation.

**1.1. Genome-wide DNA methylation data**

A detailed description of the laboratory methods and quality control applied for the DNA methylation data within each cohort can be seen in (1). In short, DNA was isolated from buffy coat by applying the salt precipitation method, and the EZ Methylation Gold kit (Zymo Research, Orange County, CA, United States) was used to bisulfite convert 500 ng DNA per sample. The Infinium HumanMethylation450K BeadChip (Illumina, San Diego, CA, United States) was applied to measure the DNA methylation, and quality control (QC) was performed using a procedure put forward by (3), using the Minfi (4) and MethylAid (5) R-packages. MethylAid provides a thorough step-by-step QC, which in addition to the usual sample and probe exclusion, also makes use of the internal QC control probes present on the array (4). In the QC, sample exclusion criteria were: (1) < 95% of probes with a detection p value < 0.01, (2) samples failing the internal quality control probes of the array, or (3) inadequate verification of sex by multidimensional scaling of the X chromosome probe values. Probe exclusion criteria: (1) detection p value > 0.01, (2) a raw intensity value of zero, (3) low bead count (< 3 beads), (4) cross-relative probes (as defined in (6)) and, (5) measurement success rate < 95%. Normalization was done by applying functional normalization (7), which is a principal component analysis (PCA) based method known to handle technical variation well, among others by the use of the between array normalization control probes on the array (7). Lastly, in the procedure (3), a PCA is performed after data processing including technical variables as well as covariates relevant for the phenotype of interest. The purpose is to identify the PCs describing the majority of the variance in the methylation data, and subsequently include either the PC or a co-variate correlated to the PC in the statistical model. As such, this approach enables inspection and correction for potential remaining technical variation.

**1.2. Genome-wide transcriptomic data**

A detailed description of the laboratory methods and quality control applied for the transcriptomic data in each cohort can be seen in (2). Briefly, PAXgene Blood RNA Tubes (PreAnalytiX GmbH, Hombrechtikon, Switzerland) was used to collect whole blood samples. These samples were collected at the same point as the blood samples used for the DNA methylation analyses, as described above. The PAXgene Blood miRNA kit (QIAGEN) was applied to extract total RNA, and the Agilent SurePrint G3 Human GE 8 × 60K Microarray version 1, 2 or 3 (Agilent Technologies) was used to examine gene expression. The ‘Two-Color Microarray-Based Gene Expression Analysis – Low Input Quick Amp Labeling’ protocol (Agilent Technologies) was used for sample labeling and array hybridization; a reference of pooled samples was labeled with Cy3, while samples were labeled with Cy5. For array image analysis the Agilent Feature Extraction software v. 10.7.3.1 (Agilent technologies) was applied. For background correction of the raw intensity, the normexp method was used within-array normalization by Loess normalization method, and between-array normalization by quantile normalization in the limma R-package (8), the two latter with the purpose to lower potential technical variation. Afterwards, the obtained data was applied to calculate log2-transformed Cy5/Cy3 ratios. K-nearest neighbors averaging was used to impute missing values, and replicate probes were collapsed by calculation of the median. Similarly to the DNA methylation data, a PCA was performed for the gene expression data for identifying relevant covariates or PCs for inclusion in the statistical models, also with the aim to correct for potential remaining technical variation.

**2. Imputation of co-variates with missing data**

Smoking status was based on self-report and was available for all but one (0.1%) individual out of the 835 individuals. This individual was set to be a never smoker, as the majority of the study population was never smokers. Furthermore, systolic blood pressure and blood lipid levels had been measured in parts of the study population. Systolic blood pressure measurements of the upper right arm had been performed as a standard resting measurement sitting (measured twice with a one-minute break). The two blood pressure measurements displayed very similar distributions (data not shown), yet due to missingness in the second measurement, the first one was used in the present study. Such blood pressure value was missing for 95 (11%) out of 835 individuals. Finally, blood lipid levels, measured by routine hospital measurements, were missing for 478 (57%) out of the 835 individuals.

As the missingness of systolic blood pressure and blood lipid levels was per survey (i.e., blood pressure had been measured in the LifeSpan and MADT cohorts, but not in the LSADT cohort, and blood lipid levels had been measured in the LifeSpan and LSADT cohorts, yet not in the MADT cohort), the missingness is considered missing as random (MAR). Hence, imputation was performed using Multiple Imputation by Chain Equations (MICE) method, known for its excellent abilities regarding imputation of values MAR (9). MICE generates multiple samples of imputed datasets, where the variability across the multiple imputed datasets captures the statistical uncertainty in data imputations. MICE estimates missing values of individual variables using observed values of other variables, where the imputations are repeated for a number of cycles, ideally until the imputed data converge. This process is performed for several datasets to improve power. Finally, the association analysis of interest (in the present study EWAS or TWAS) is performed for all the datasets resulting in multiple results and, the results are pooled by use of Rubin’s rules. For further details see (9) .

In the current study, 20 cycles were conducted to create 20 datasets for each statistical analysis (EWAS or TWAS), where predictive mean matching was selected as the imputation method. In order to define the variables used for imputation in the 20 cycles, the correlation was initially investigated between the real nonHDL values, respectively the real systolic blood pressure values, and all the survey and register data included in the present study, as well as the omics data. The investigation of correlation was done separately for the genome-wide DNA methylation data and the genome-wide gene expression data, and separately for nonHDL and systolic blood pressure. This separation entailed that the variables later used for imputation was specific for nonHDL and systolic blood pressure within either analysis of genome-wide DNA methylation data or genome-wide gene expression data. The top fifty variables correlating the most to nonHDL, respectively systemic blood pressure, were chosen for imputation of either nonHDL or systemic blood pressure. In the present dataset it was first of all omics variables (i.e., CpGs values for the DNA methylation data and probes for the gene expression data), which were among the top fifty variables for nonHDL, respectively systolic blood pressure (data not shown). In addition, sensitivity analyses were performed in the individuals holding real nonHDL and systolic blood pressure values: EWAS/TWAS analyses with or without inclusion of the two variables (i.e., nonHDL and systolic blood pressure) led to the same overall conclusions (data not shown). Including either the real values or the imputed values for the two variables also led to the same conclusions (data not shown). That blood lipid levels, and blood pressure did not appear to be important confounders in the present data, was also reflected in the principal component analysis (PCA), where neither variable correlated highly to the variance in the genome-wide biological data (see below). The systolic blood pressure and blood lipid levels did not correlate well to the CVD outcome variables either (data not shown).

Finally, in the present study we also tested the algorithm presented by McCartney (10) for estimating blood lipid levels based on genome-wide DNA methylation data. Yet, the correlation between the estimated values and real values was modest in the present data (data not shown). To the best of our knowledge no other algorithms for estimating blood lipid levels or systolic blood pressure based on epigenetic data had been published at the time of conducting the present study.

**3. Principal component analysis**

For both types of omics data, principal component analysis (PCA) was initially performed (similarly to (1)) of all survey and register data considered in the present study, and the DNA methylation, respectively, the gene expression data, with the aim to examine the correlation between potential confounder variables and the PCs of the omics data. Subsequently, either the confounder variable or the PC was included in the statistical analysis. The PCA showed that the majority of the variance in the omics data was covered by the top three PCs, as the subsequent PCs accounted for less than 7% of the variance for the gene expression data and less than 8% for the epigenetic data (data not shown). Consequently, only PC1, PC2 and PC3 were considered further. Subsequently, the correlation between PCs 1-3 of the epigenetic data, respectively the PCs 1-3 of the gene expression data, and the potential confounders of the survey data was inspected. For the DNA methylation data, PC1 correlated highly with sex (correlation coefficient = -0.99) and PC2 correlated with age (correlation coefficient = -0.79), and as sex and age were to be included as covariate in the statistical analysis, only PC3 was included in the statistical analyses. PC3 of the epigenetic data correlated with cell counts (correlation coefficient = -0.66 and 0.66 for lymphocytes and neutrophils, respectively). For the gene expression data, PC2 was included in the statistical analyses, as it (similarly to PC3 for the epigenetic data) correlated with cell counts (correlation coefficient = -0.65 and 0.63 for lymphocytes and neutrophils, respectively). PC1 and PC3 of the gene expression data correlated moderately with age (correlation coefficient = -0.42 and 0.27, respectively). As PC1 covered more of the variance in the gene expression data than PC3, TWAS with and without the inclusion of PC1 was initially performed as a sensitivity analysis. Here, inclusion or omission of PC1 showed no impact on the results (data not shown) and was therefore not included in the final models. For consistency with the statistical models applied for the two types of omics data, and in order not to induce collinearity issues, PC3 was left out of the analysis the gene expression data. Consequently, the statistical models of the present project included the following co-variates: both EWAS and TWAS: age at blood sampling, sex, nonHDL, systolic blood pressure and smoking status. Moreover, the statistical models include PC3 for EWAS and PC2 for TWAS, both reflecting cell counts. Moreover, in the individual level analysis an omics dataset variable, as well as twin pair ID, were considered in order to take batch effect, respectively the dependency between the co-twins in the twin pairs, into account. These two variables were not considered in the twin pair level analysis, as all twin pairs were analyzed on the same omics array and within the same omics dataset (i.e., there is no variation in the dataset variable within the twin pairs), and as the dependency between the co-twins in a pair is not relevant in the intra-pair analysis.

References

1. Soerensen M, Li W, Debrabant B, Nygaard M, Mengel-From J, Frost M, et al. Epigenome-wide exploratory study of monozygotic twins suggests differentially methylated regions to associate with hand grip strength. Biogerontology. 2019;20(5):627-47.

2. Nygaard M, Larsen MJ, Thomassen M, McGue M, Christensen K, Tan Q, et al. Global expression profiling of cognitive level and decline in middle-aged monozygotic twins. Neurobiology of Aging. 2019;84:141-7.

3. Tobi EW, Slieker RC, Stein AD, Suchiman HE, Slagboom PE, van Zwet EW, et al. Early gestation as the critical time-window for changes in the prenatal environment to affect the adult human blood methylome. Int J Epidemiol. 2015;44(4):1211-23.

4. Aryee MJ, Jaffe AE, Corrada-Bravo H, Ladd-Acosta C, Feinberg AP, Hansen KD, et al. Minfi: a flexible and comprehensive Bioconductor package for the analysis of Infinium DNA methylation microarrays. Bioinformatics. 2014;30(10):1363-9.

5. van Iterson M, Tobi EW, Slieker RC, den Hollander W, Luijk R, Slagboom PE, et al. MethylAid: visual and interactive quality control of large Illumina 450k datasets. Bioinformatics. 2014;30(23):3435-7.

6. Chen YA, Lemire M, Choufani S, Butcher DT, Grafodatskaya D, Zanke BW, et al. Discovery of cross-reactive probes and polymorphic CpGs in the Illumina Infinium HumanMethylation450 microarray. Epigenetics. 2013;8(2):203-9.

7. Fortin J-P, Labbe A, Lemire M, Zanke BW, Hudson TJ, Fertig EJ, et al. Functional normalization of 450k methylation array data improves replication in large cancer studies. Genome Biology. 2014;15(11):503.

8. Ritchie ME, Silver J, Oshlack A, Holmes M, Diyagama D, Holloway A, et al. A comparison of background correction methods for two-colour microarrays. Bioinformatics. 2007;23(20):2700-7.

9. Azur MJ, Stuart EA, Frangakis C, Leaf PJ. Multiple imputation by chained equations: what is it and how does it work? Int J Methods Psychiatr Res. 2011;20(1):40-9.

10. McCartney DL, Hillary RF, Stevenson AJ, Ritchie SJ, Walker RM, Zhang Q, et al. Epigenetic prediction of complex traits and death. Genome Biology. 2018;19(1):136.
