# Supplementary material for: Multi-omics association study of DNA methylation and gene expression levels and diagnoses of cardiovascular diseases in Danish Twins
Source: Clin Epigenetics. 2024 Aug 26;16:117. doi: 10.1186/s13148-024-01727-6 (PMC11348607; doi:10.1186/s13148-024-01727-6)
Supplement: Supplementary file 6 — Additional file 6. Supplementary Figures. [file 13148_2024_1727_MOESM6_ESM.zip › Supplementary Figures/Figure caption.docx]

**Supplementary Information**

Supplementary Figures (Volcano plots): Supplementary Figures.pdf.

**Notes: Volcano plots** for each analysis. Genes colored red represents overlapping genes with a p value below 0.05 identified in both individual-level analysis and twin pair level analyses (e.g. found in the b1 or b2 overlap in figure 1). Genes colored green represents overlapping genes with a p value below 0.05 identified in both prevalent analyses and incident analyses (e.g. found in c1 overlap in figure 1).
