# Supplementary figures and images for: Multi-omics association study of DNA methylation and gene expression levels and diagnoses of cardiovascular diseases in Danish Twins
Source: Clin Epigenetics. 2024 Aug 26;16:117. doi: 10.1186/s13148-024-01727-6 (PMC11348607; doi:10.1186/s13148-024-01727-6)

# Incident Individual Analysis

## Arterial and Other Cardiovascular Diseases: EWAS

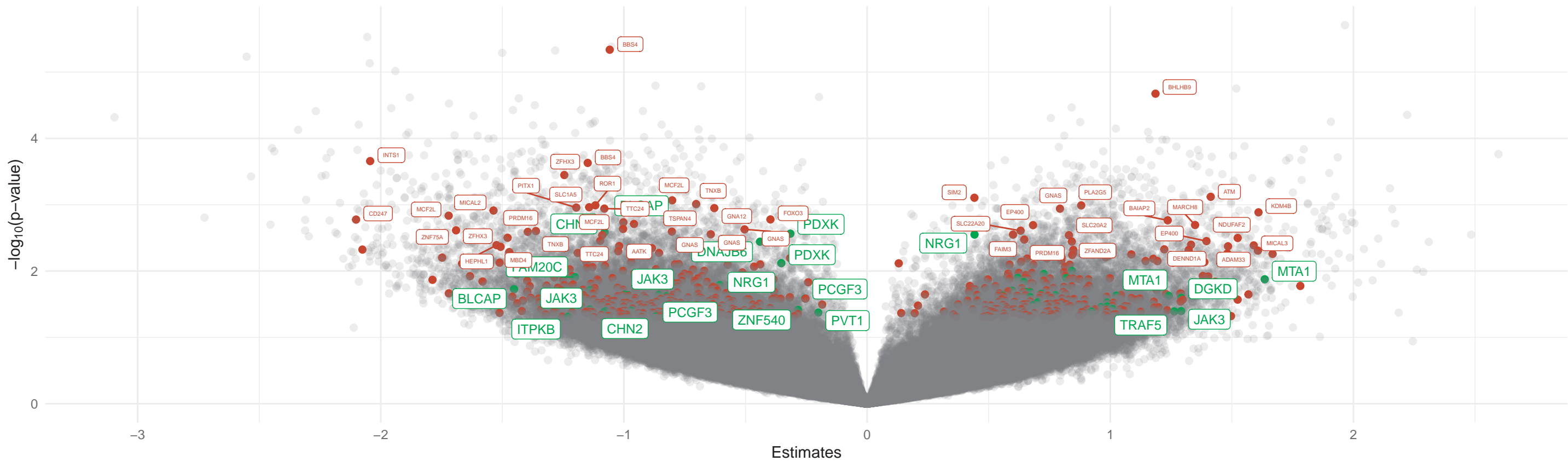

Supplement: Supplementary file 6 — Additional file 6. Supplementary Figures. [file 13148_2024_1727_MOESM6_ESM.zip › Supplementary Figures/Arterial and Other Cardiovascular Diseases - Incident Individual Analysis EWAS.pdf]

# Incident Individual Analysis

## Arterial and Other Cardiovascular Diseases: TWAS

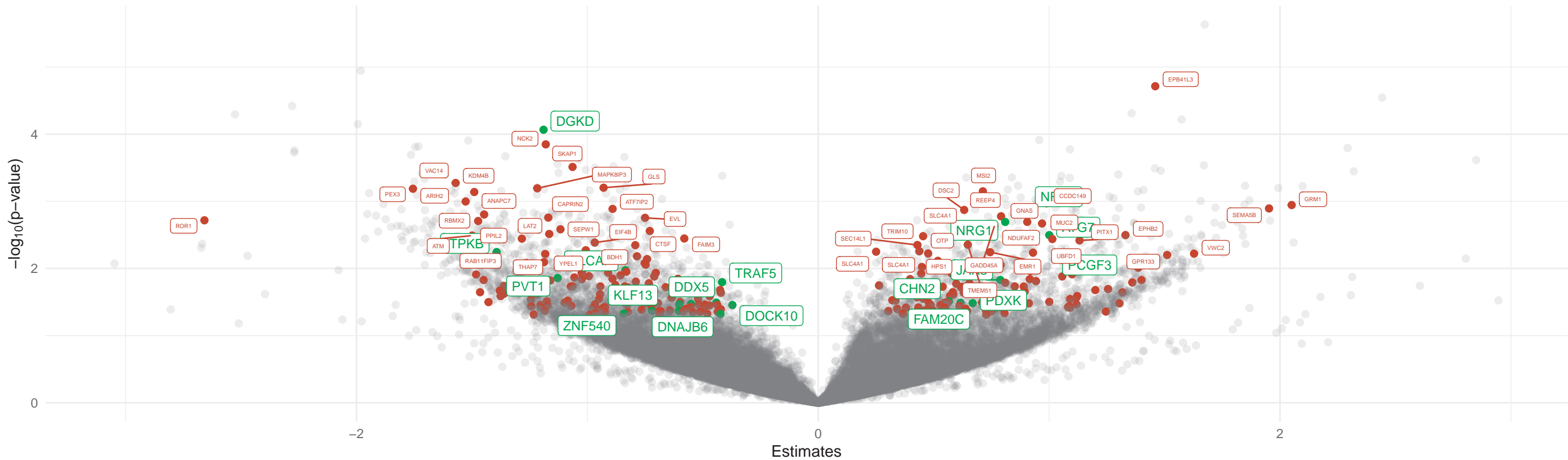

Supplement: Supplementary file 6 — Additional file 6. Supplementary Figures. [file 13148_2024_1727_MOESM6_ESM.zip › Supplementary Figures/Arterial and Other Cardiovascular Diseases - Incident Individual Analysis TWAS.pdf]

# Incident Twinpair Analysis

## Arterial and Other Cardiovascular Diseases: EWAS

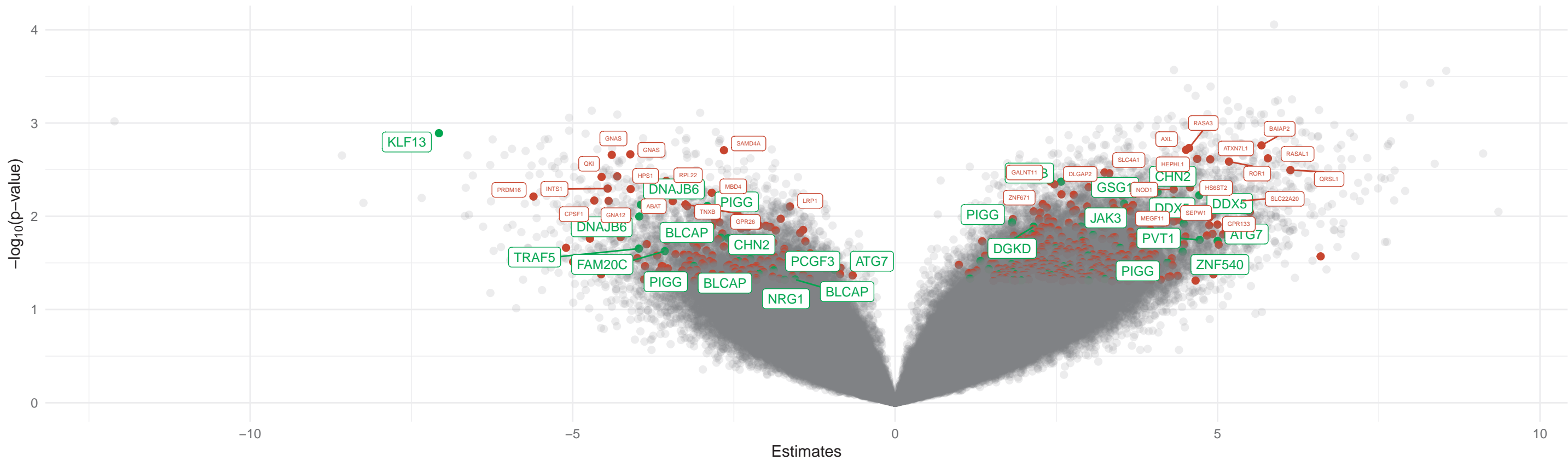

Supplement: Supplementary file 6 — Additional file 6. Supplementary Figures. [file 13148_2024_1727_MOESM6_ESM.zip › Supplementary Figures/Arterial and Other Cardiovascular Diseases - Incident Twinpair Analysis EWAS.pdf]

# Incident Twinpair Analysis

## Arterial and Other Cardiovascular Diseases: TWAS

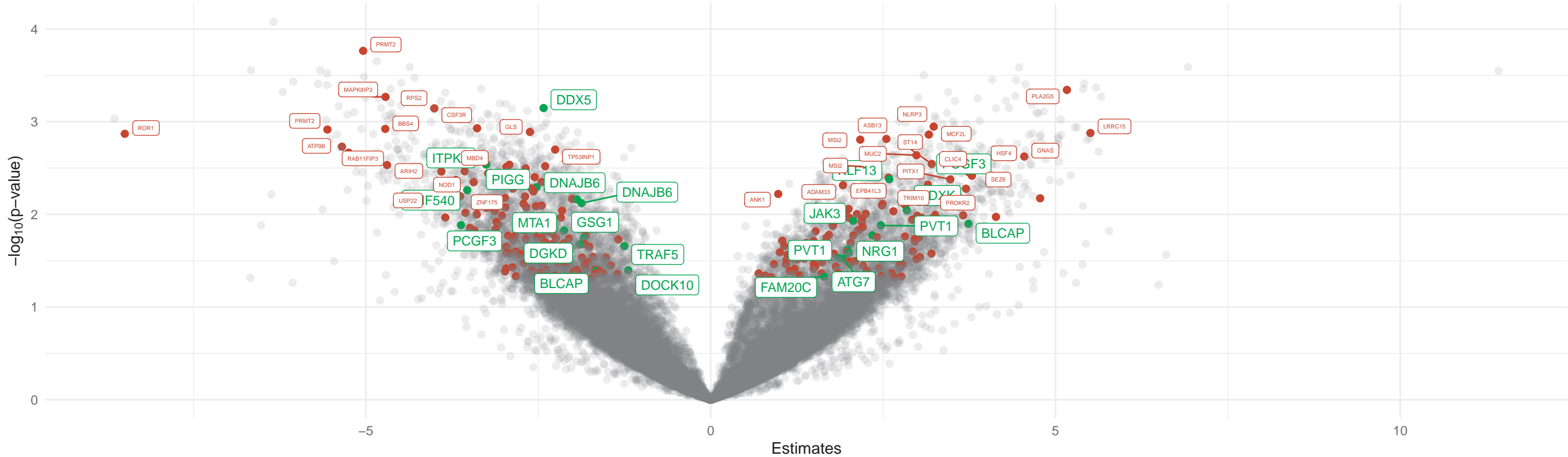

Supplement: Supplementary file 6 — Additional file 6. Supplementary Figures. [file 13148_2024_1727_MOESM6_ESM.zip › Supplementary Figures/Arterial and Other Cardiovascular Diseases - Incident Twinpair Analysis TWAS.pdf]

# Prevalent Individual Analysis

## Arterial and Other Cardiovascular Diseases: EWAS

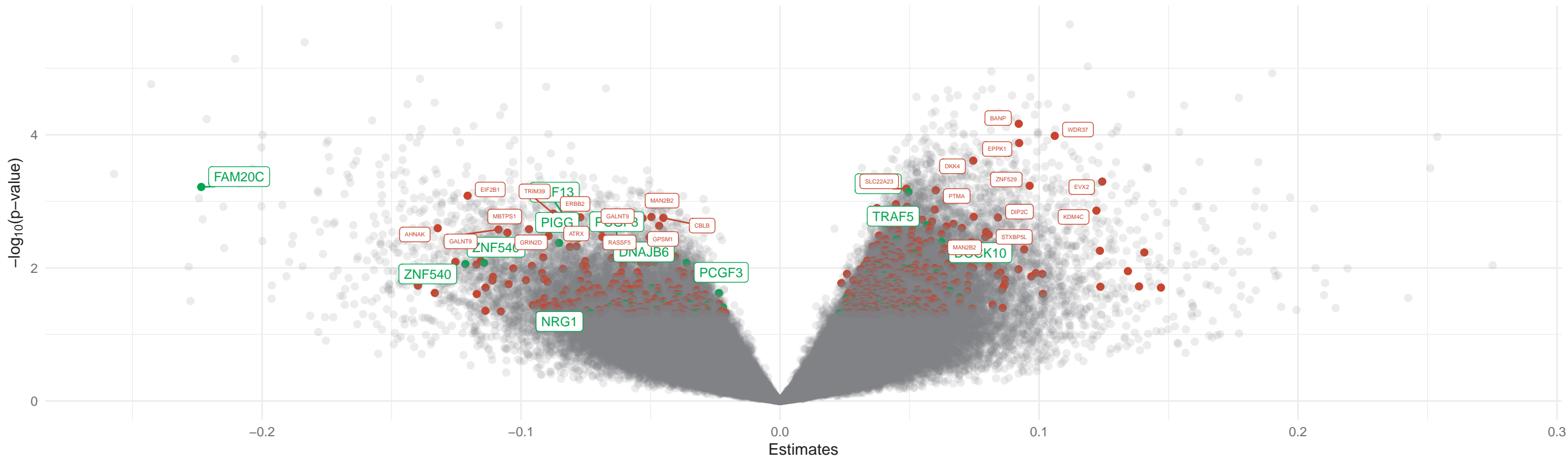

Supplement: Supplementary file 6 — Additional file 6. Supplementary Figures. [file 13148_2024_1727_MOESM6_ESM.zip › Supplementary Figures/Arterial and Other Cardiovascular Diseases - Prevalent Individual Analysis EWAS.pdf]

# Prevalent Individual Analysis

## Arterial and Other Cardiovascular Diseases: TWAS

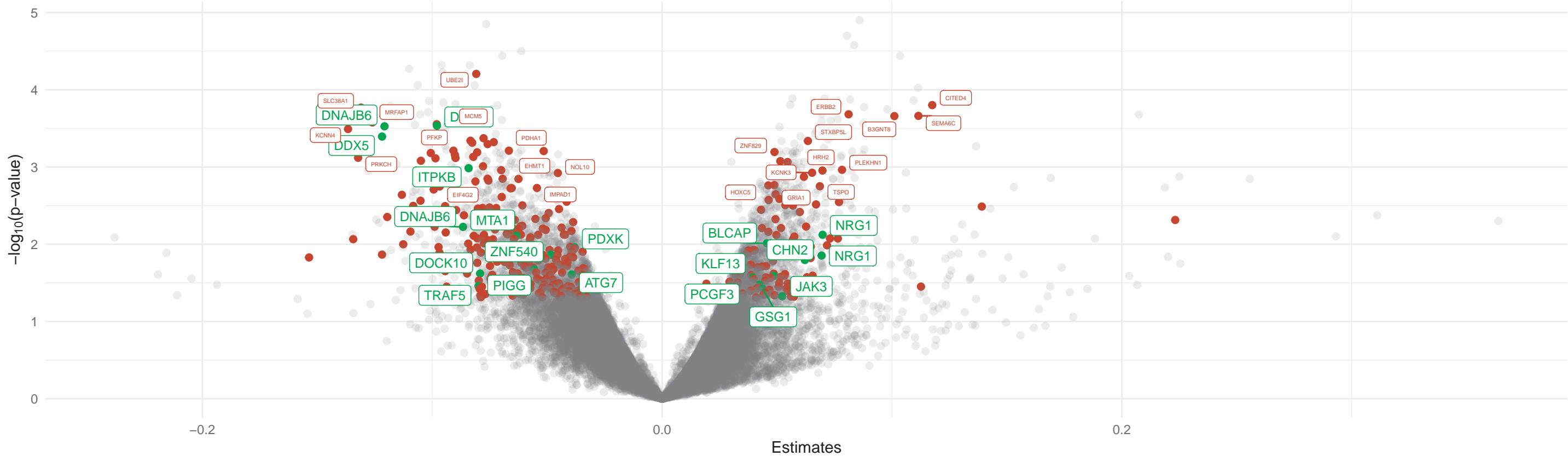

Supplement: Supplementary file 6 — Additional file 6. Supplementary Figures. [file 13148_2024_1727_MOESM6_ESM.zip › Supplementary Figures/Arterial and Other Cardiovascular Diseases - Prevalent Individual Analysis TWAS.pdf]

# Prevalent Twinpair Analysis

## Arterial and Other Cardiovascular Diseases: EWAS

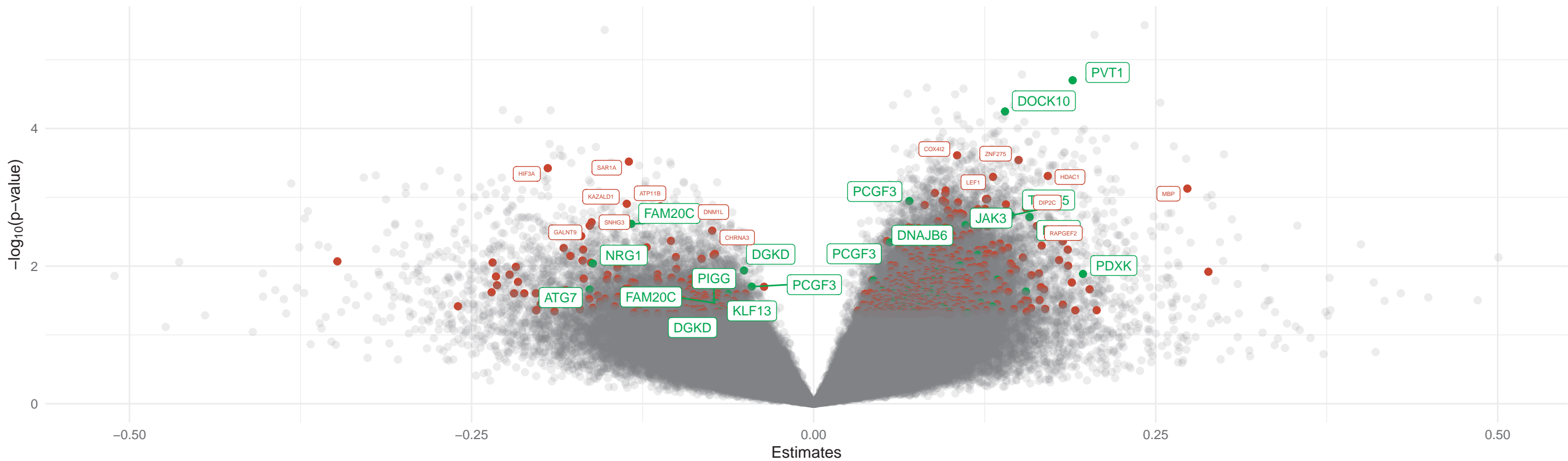

Supplement: Supplementary file 6 — Additional file 6. Supplementary Figures. [file 13148_2024_1727_MOESM6_ESM.zip › Supplementary Figures/Arterial and Other Cardiovascular Diseases - Prevalent Twinpair Analysis EWAS.pdf]

# Prevalent Twinpair Analysis

## Arterial and Other Cardiovascular Diseases: TWAS

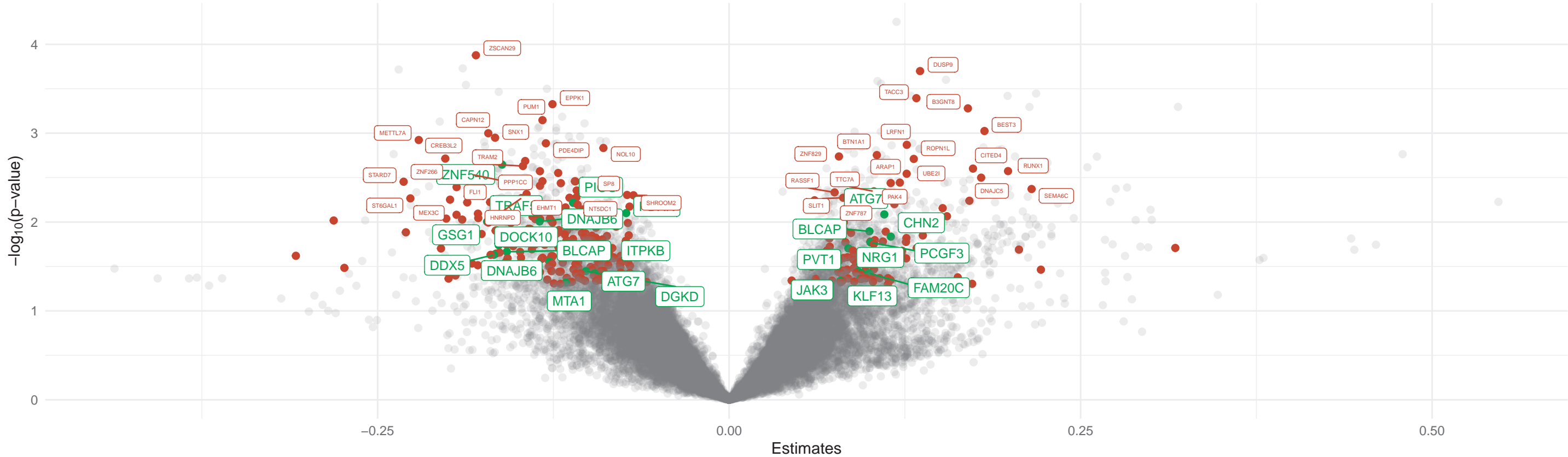

Supplement: Supplementary file 6 — Additional file 6. Supplementary Figures. [file 13148_2024_1727_MOESM6_ESM.zip › Supplementary Figures/Arterial and Other Cardiovascular Diseases - Prevalent Twinpair Analysis TWAS.pdf]

# Incident Individual Analysis

## Cerebrovascular Diseases: EWAS

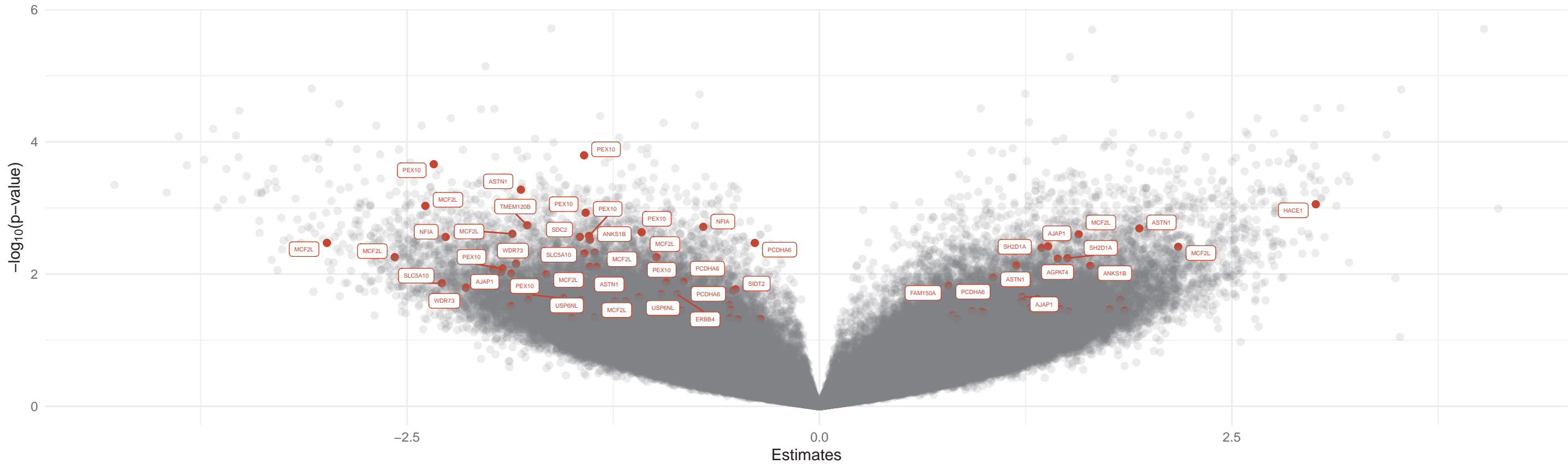

Supplement: Supplementary file 6 — Additional file 6. Supplementary Figures. [file 13148_2024_1727_MOESM6_ESM.zip › Supplementary Figures/Cerebrovascular Diseases - Incident Individual Analysis EWAS.pdf]

# Incident Individual Analysis

## Cerebrovascular Diseases: TWAS

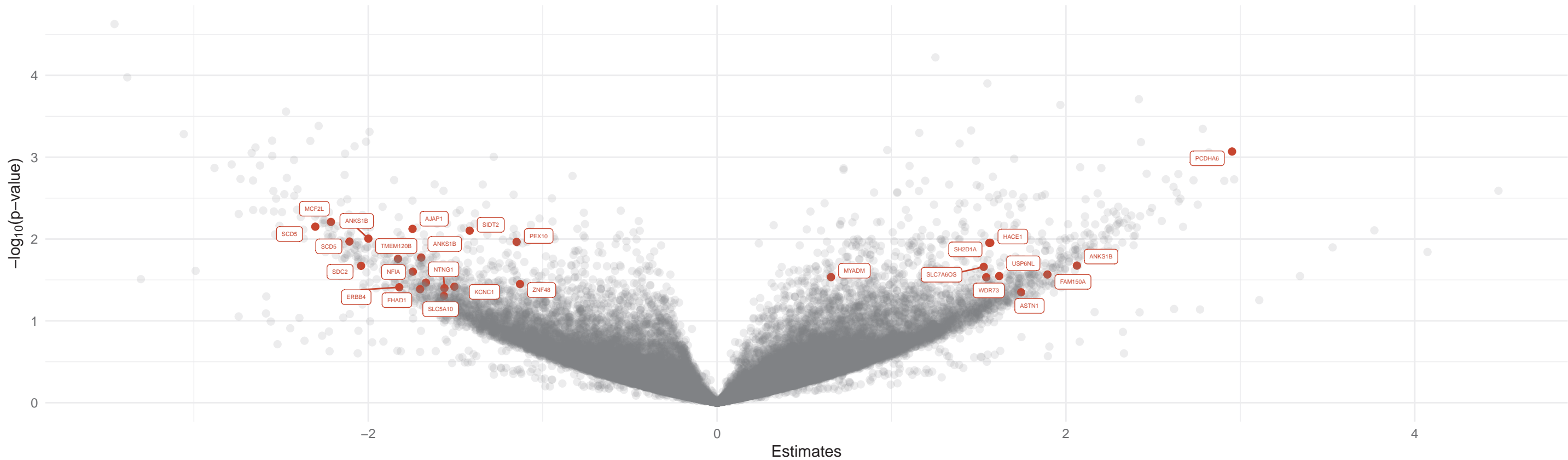

Supplement: Supplementary file 6 — Additional file 6. Supplementary Figures. [file 13148_2024_1727_MOESM6_ESM.zip › Supplementary Figures/Cerebrovascular Diseases - Incident Individual Analysis TWAS.pdf]

# Incident Twinpair Analysis Cerebrovascular Diseases: EWAS

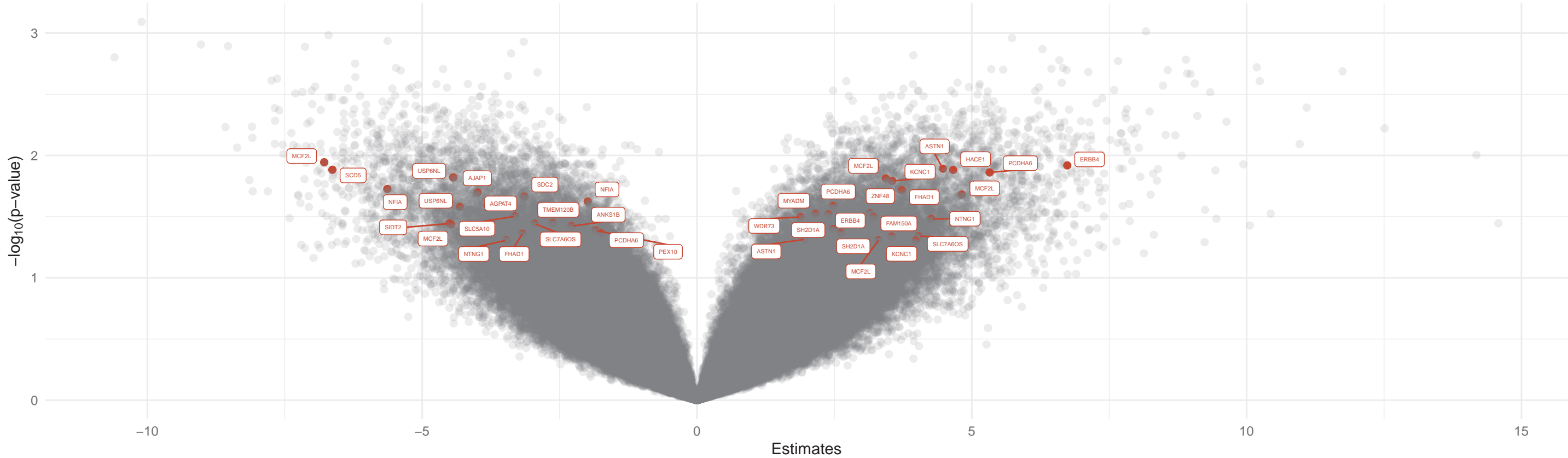

Supplement: Supplementary file 6 — Additional file 6. Supplementary Figures. [file 13148_2024_1727_MOESM6_ESM.zip › Supplementary Figures/Cerebrovascular Diseases - Incident Twinpair Analysis EWAS.pdf]

# Incident Twinpair Analysis

## Cerebrovascular Diseases: TWAS

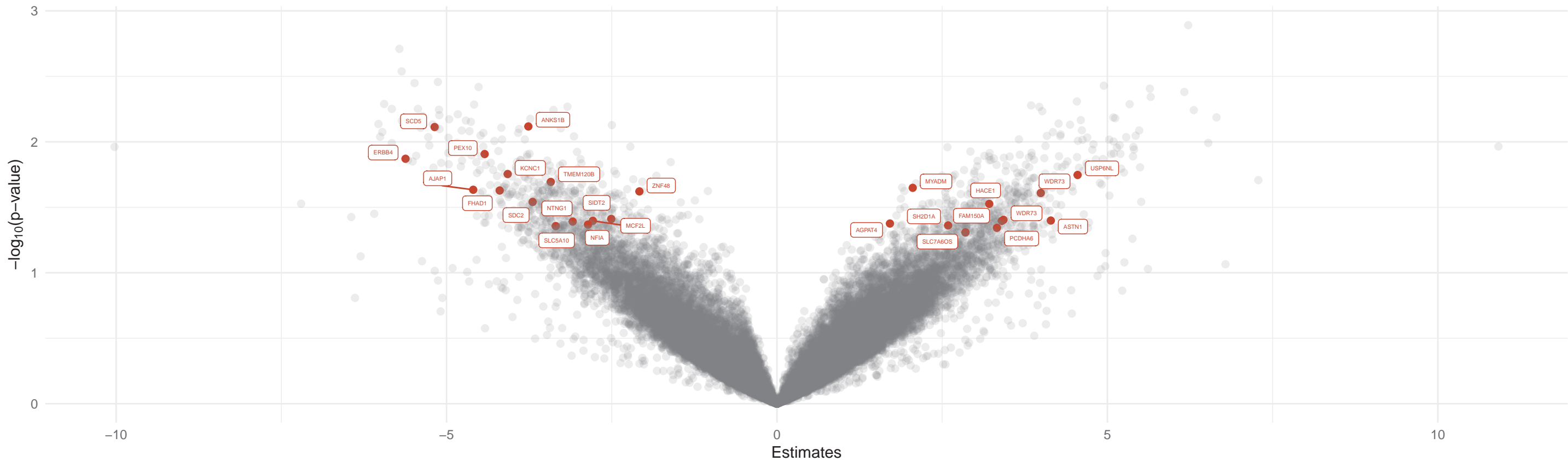

Supplement: Supplementary file 6 — Additional file 6. Supplementary Figures. [file 13148_2024_1727_MOESM6_ESM.zip › Supplementary Figures/Cerebrovascular Diseases - Incident Twinpair Analysis TWAS.pdf]

# Prevalent Individual Analysis Cerebrovascular Diseases: EWAS

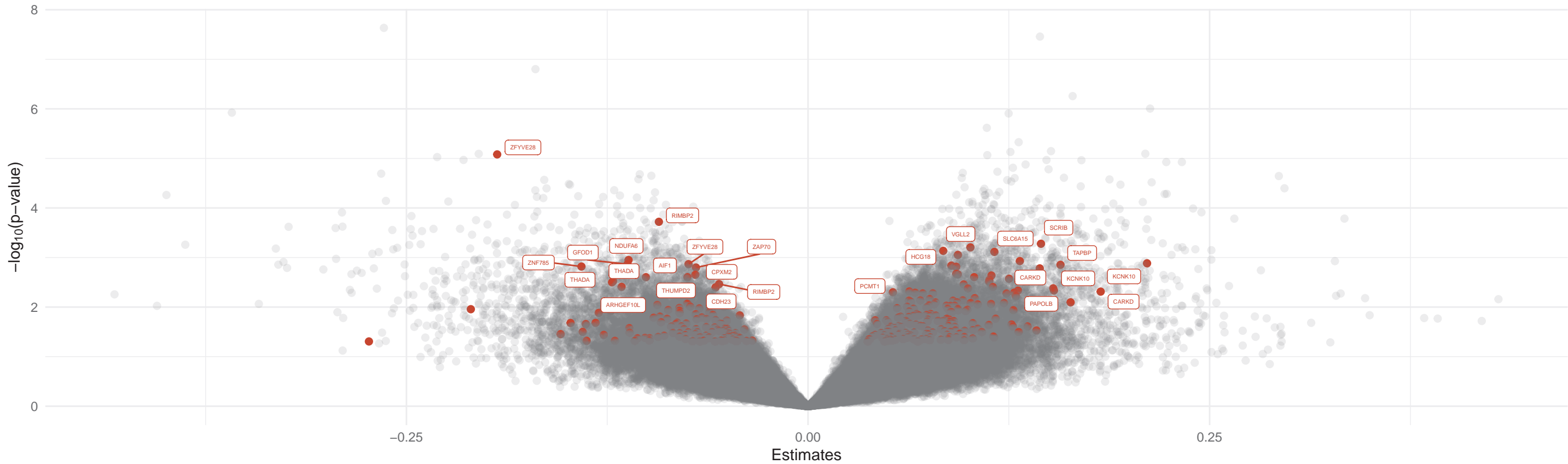

Supplement: Supplementary file 6 — Additional file 6. Supplementary Figures. [file 13148_2024_1727_MOESM6_ESM.zip › Supplementary Figures/Cerebrovascular Diseases - Prevalent Individual Analysis EWAS.pdf]

# Prevalent Individual Analysis Cerebrovascular Diseases: TWAS

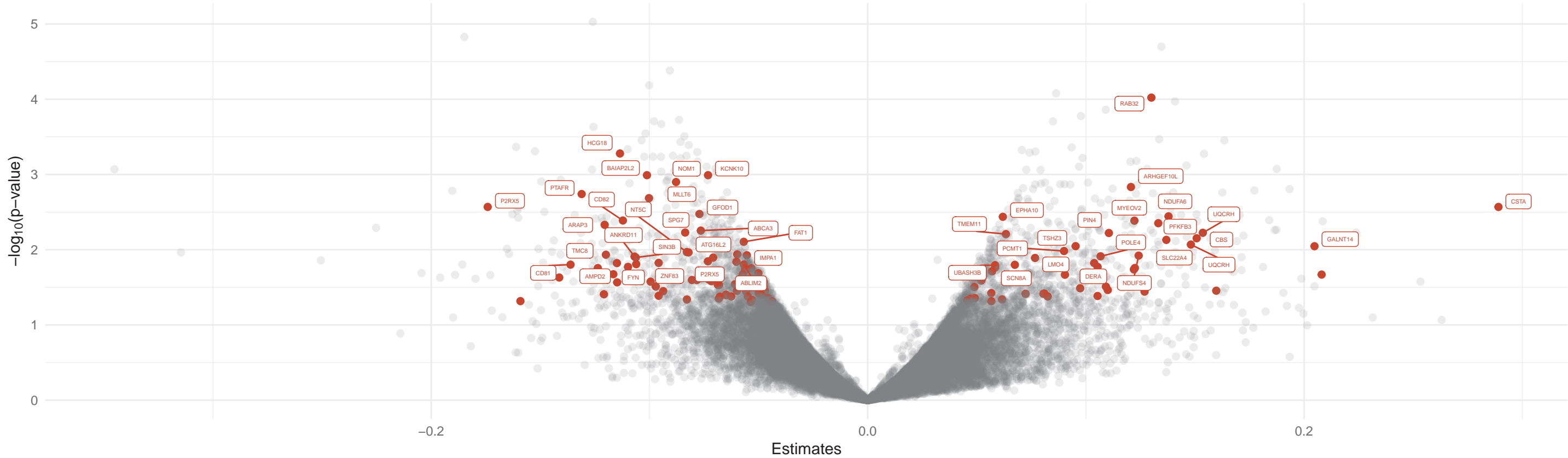

Supplement: Supplementary file 6 — Additional file 6. Supplementary Figures. [file 13148_2024_1727_MOESM6_ESM.zip › Supplementary Figures/Cerebrovascular Diseases - Prevalent Individual Analysis TWAS.pdf]

# Prevalent Twinpair Analysis

## Cerebrovascular Diseases: TWAS

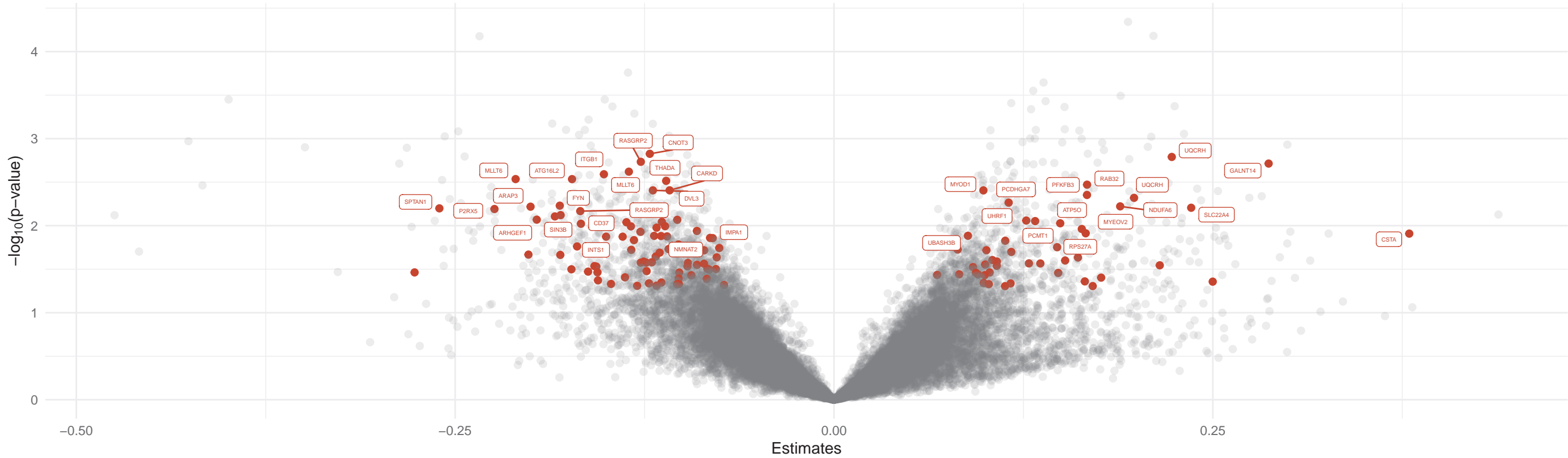

Supplement: Supplementary file 6 — Additional file 6. Supplementary Figures. [file 13148_2024_1727_MOESM6_ESM.zip › Supplementary Figures/Cerebrovascular Diseases - Prevalent Twinpair Analysis TWAS.pdf]

# Incident Individual Analysis

## Coronary Artery Diseases: TWAS

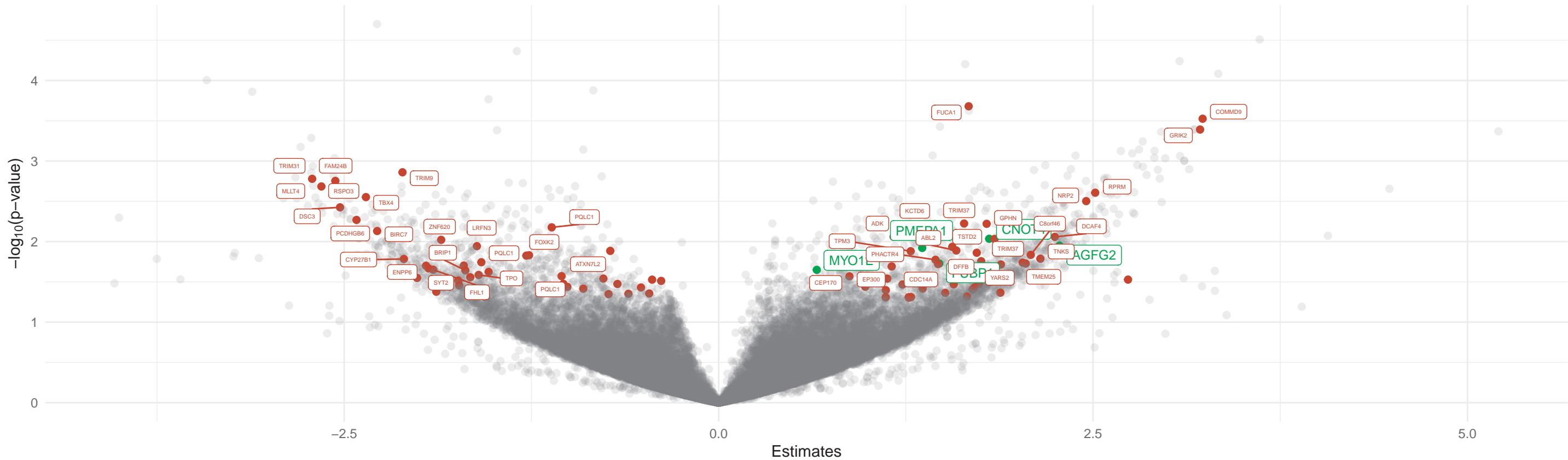

Supplement: Supplementary file 6 — Additional file 6. Supplementary Figures. [file 13148_2024_1727_MOESM6_ESM.zip › Supplementary Figures/Coronary Artery Diseases - Incident Individual Analysis TWAS.pdf]

# Incident Twinpair Analysis

## Coronary Artery Diseases: EWAS

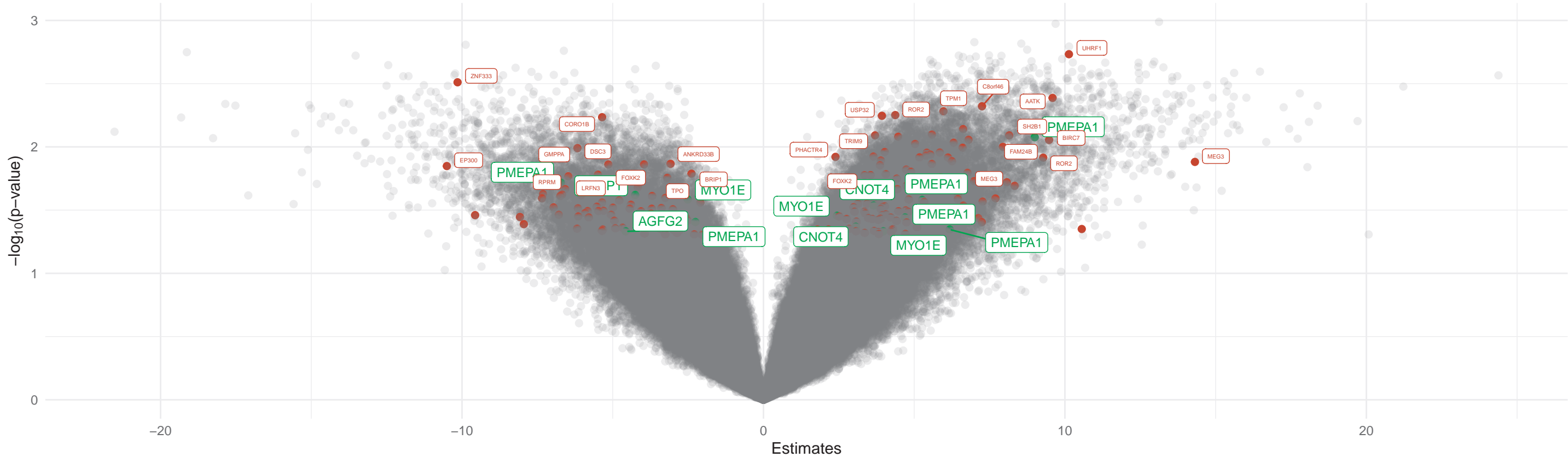

Supplement: Supplementary file 6 — Additional file 6. Supplementary Figures. [file 13148_2024_1727_MOESM6_ESM.zip › Supplementary Figures/Coronary Artery Diseases - Incident Twinpair Analysis EWAS.pdf]

# Incident Twinpair Analysis

## Coronary Artery Diseases: TWAS

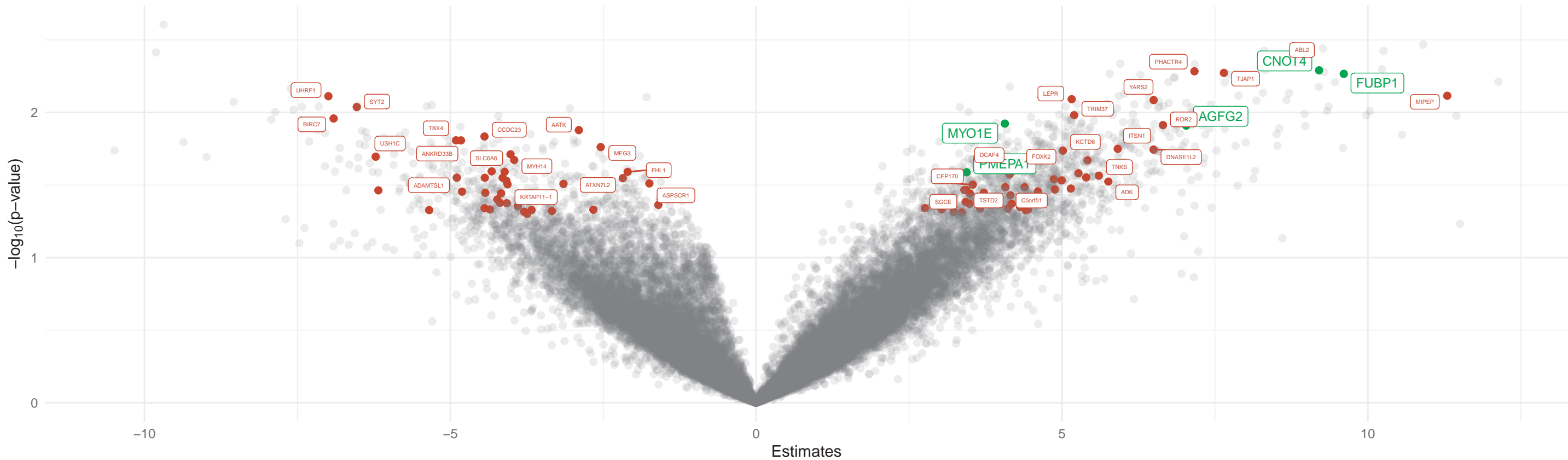

Supplement: Supplementary file 6 — Additional file 6. Supplementary Figures. [file 13148_2024_1727_MOESM6_ESM.zip › Supplementary Figures/Coronary Artery Diseases - Incident Twinpair Analysis TWAS.pdf]

# Prevalent Individual Analysis

## Coronary Artery Diseases: TWAS

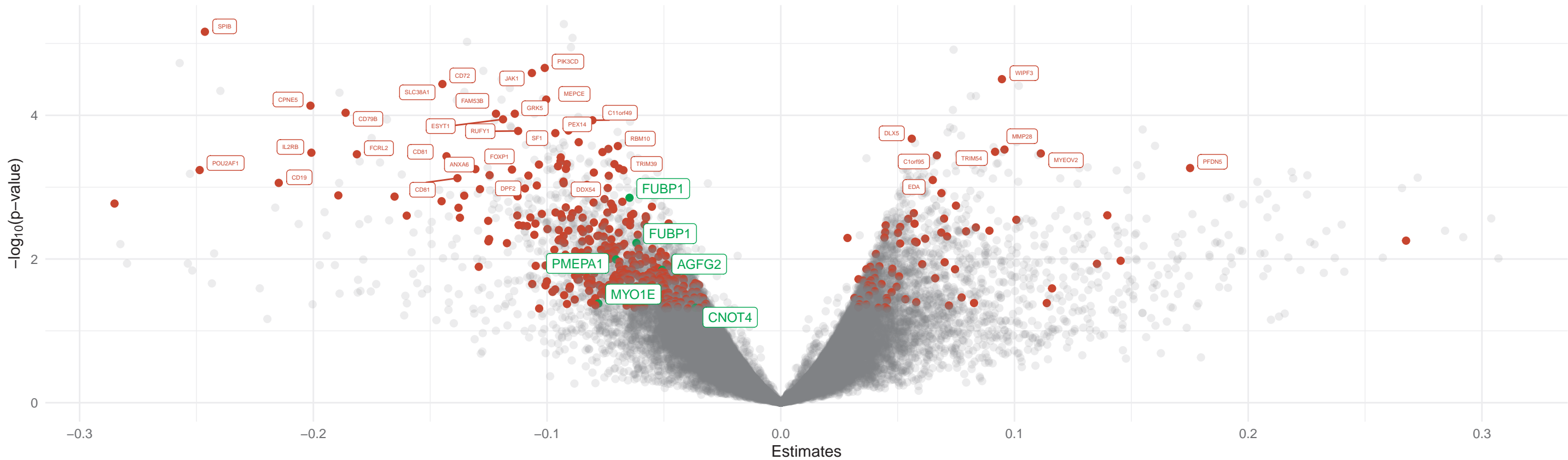

Supplement: Supplementary file 6 — Additional file 6. Supplementary Figures. [file 13148_2024_1727_MOESM6_ESM.zip › Supplementary Figures/Coronary Artery Diseases - Prevalent Individual Analysis TWAS.pdf]

# Prevalent Twinpair Analysis

## Coronary Artery Diseases: EWAS

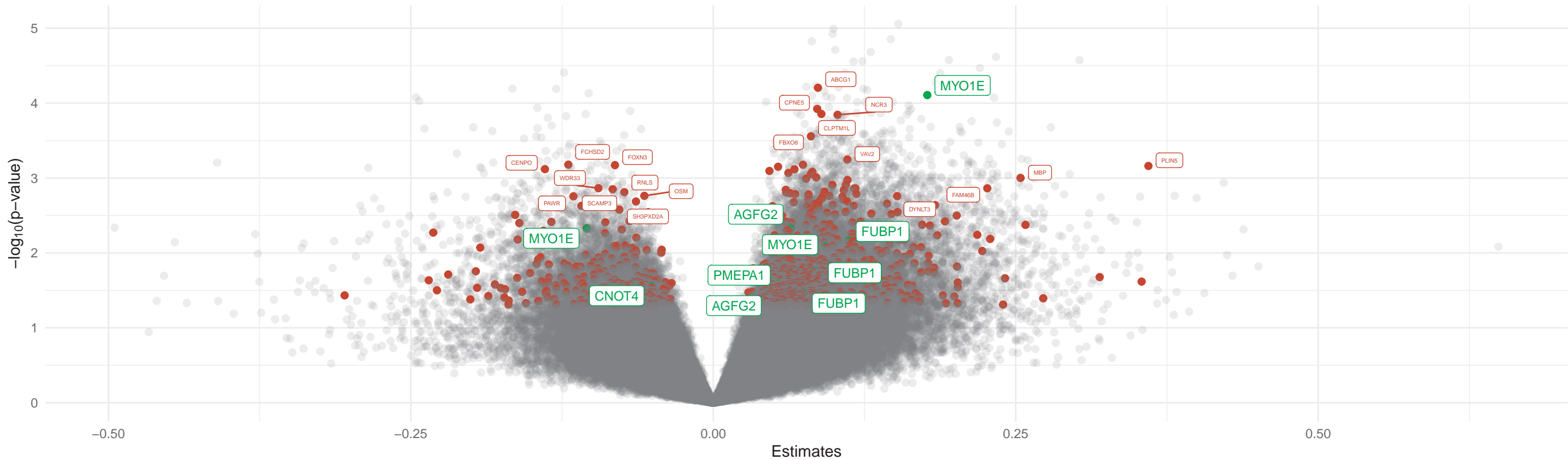

Supplement: Supplementary file 6 — Additional file 6. Supplementary Figures. [file 13148_2024_1727_MOESM6_ESM.zip › Supplementary Figures/Coronary Artery Diseases - Prevalent Twinpair Analysis EWAS.pdf]

# Prevalent Twinpair Analysis

## Coronary Artery Diseases: TWAS

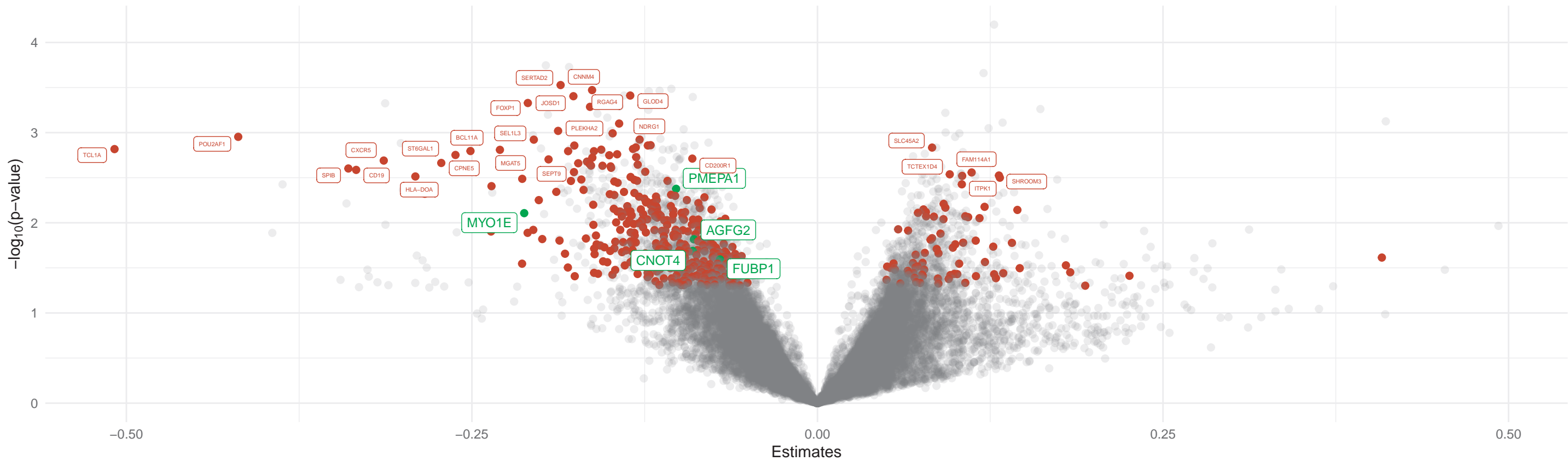

Supplement: Supplementary file 6 — Additional file 6. Supplementary Figures. [file 13148_2024_1727_MOESM6_ESM.zip › Supplementary Figures/Coronary Artery Diseases - Prevalent Twinpair Analysis TWAS.pdf]

# Incident Individual Analysis

## Diseases of the Veins and Lymphatic system: EWAS

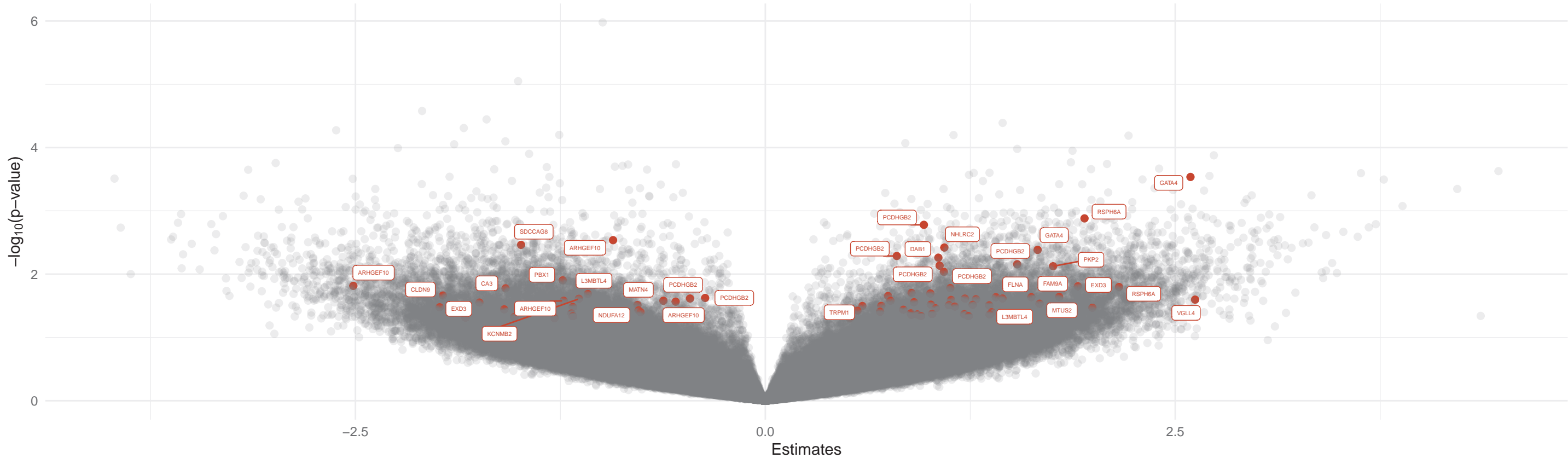

Supplement: Supplementary file 6 — Additional file 6. Supplementary Figures. [file 13148_2024_1727_MOESM6_ESM.zip › Supplementary Figures/Diseases of the Veins and Lymphatic system - Incident Individual Analysis EWAS.pdf]

# Incident Individual Analysis

## Diseases of the Veins and Lymphatic system: TWAS

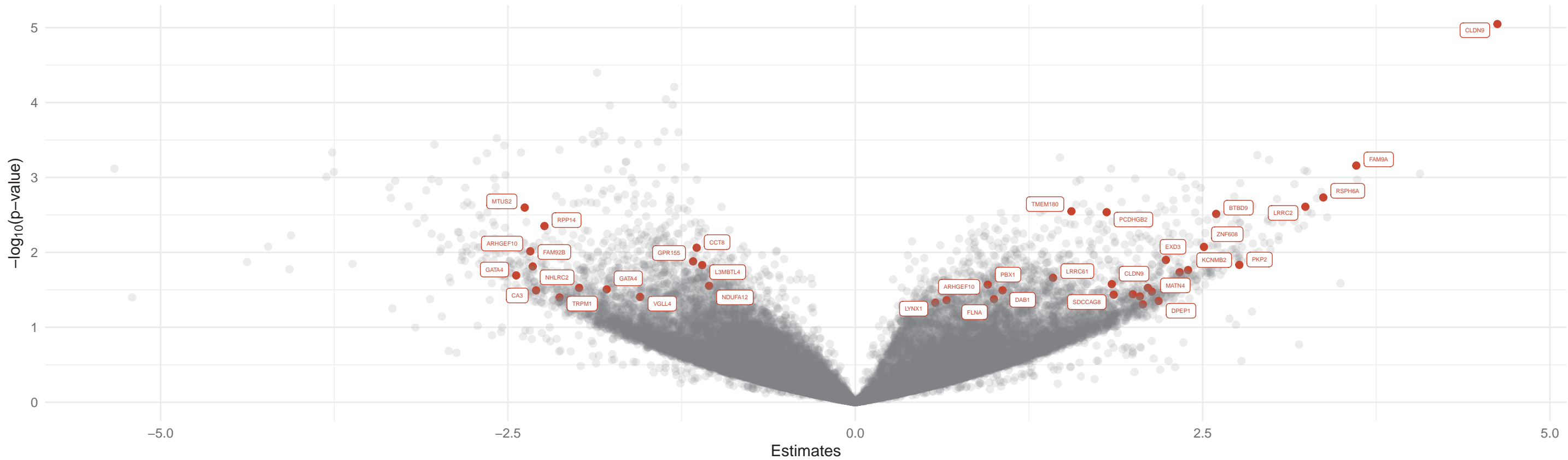

Supplement: Supplementary file 6 — Additional file 6. Supplementary Figures. [file 13148_2024_1727_MOESM6_ESM.zip › Supplementary Figures/Diseases of the Veins and Lymphatic system - Incident Individual Analysis TWAS.pdf]

# Incident Twinpair Analysis

## Diseases of the Veins and Lymphatic system: EWAS

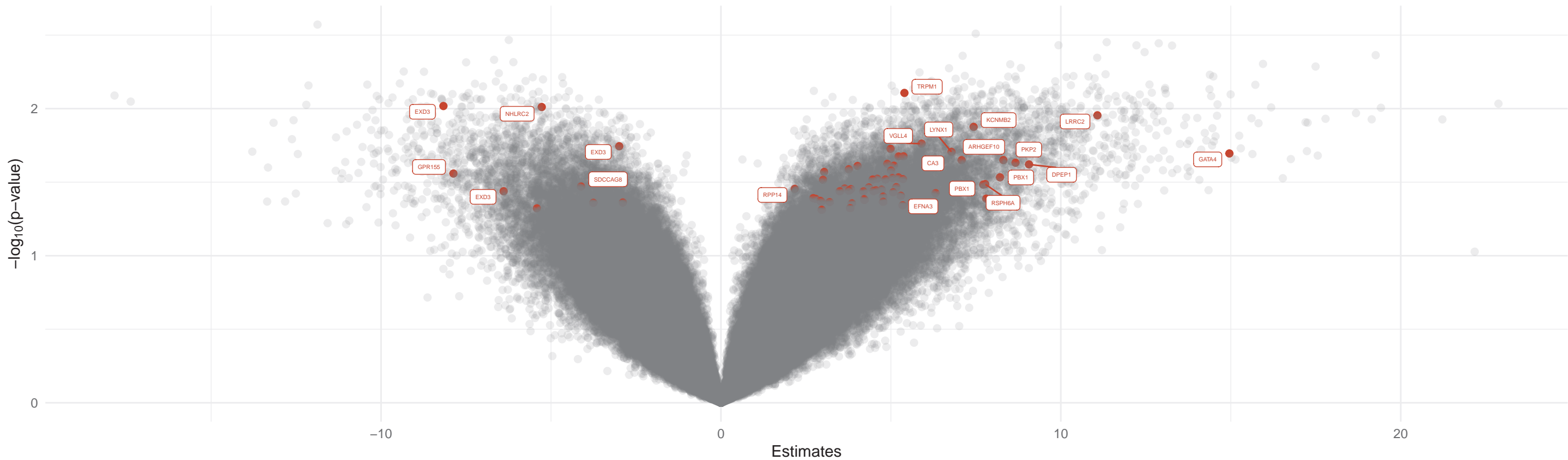

Supplement: Supplementary file 6 — Additional file 6. Supplementary Figures. [file 13148_2024_1727_MOESM6_ESM.zip › Supplementary Figures/Diseases of the Veins and Lymphatic system - Incident Twinpair Analysis EWAS.pdf]

# Incident Twinpair Analysis

## Diseases of the Veins and Lymphatic system: TWAS

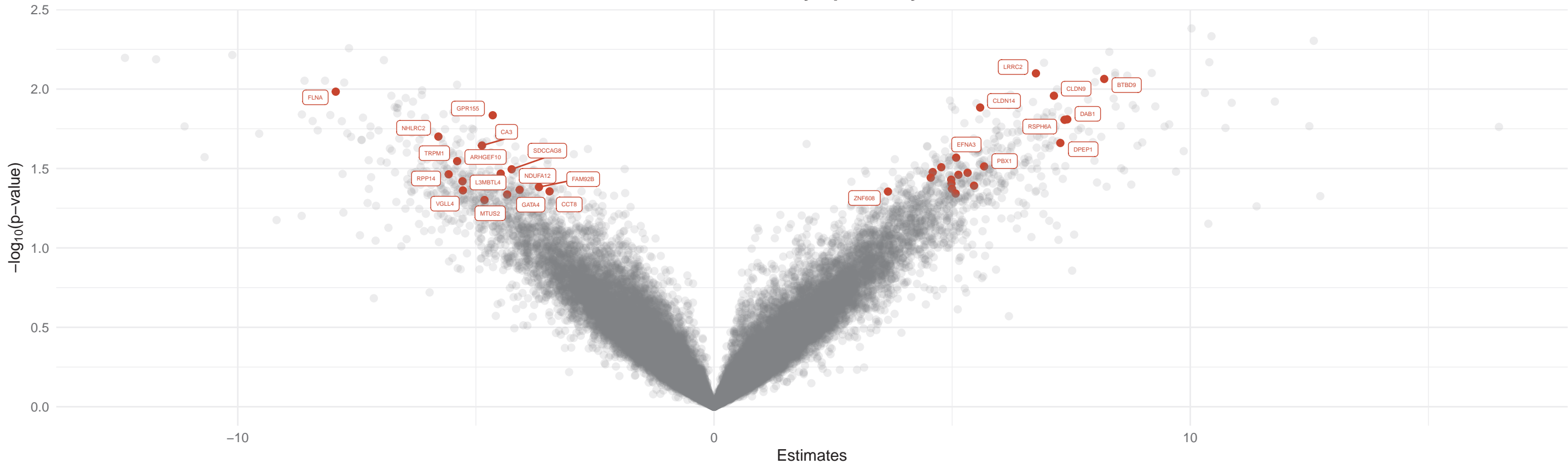

Supplement: Supplementary file 6 — Additional file 6. Supplementary Figures. [file 13148_2024_1727_MOESM6_ESM.zip › Supplementary Figures/Diseases of the Veins and Lymphatic system - Incident Twinpair Analysis TWAS.pdf]

# Prevalent Individual Analysis

## Diseases of the Veins and Lymphatic system: EWAS

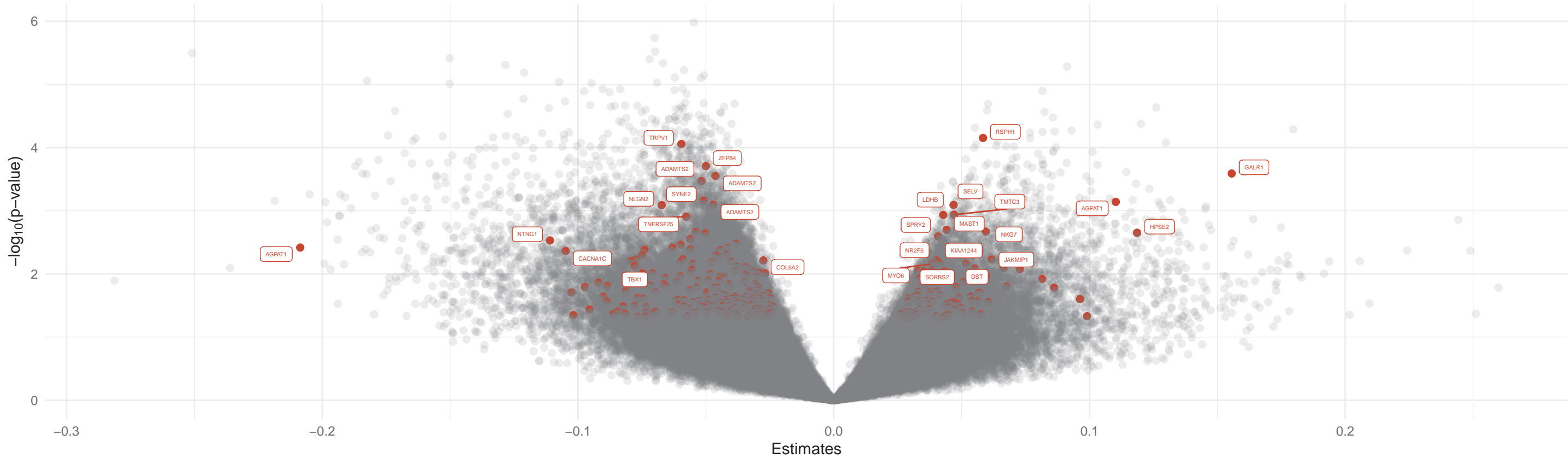

Supplement: Supplementary file 6 — Additional file 6. Supplementary Figures. [file 13148_2024_1727_MOESM6_ESM.zip › Supplementary Figures/Diseases of the Veins and Lymphatic system - Prevalent Individual Analysis EWAS.pdf]

# Prevalent Individual Analysis

## Diseases of the Veins and Lymphatic system: TWAS

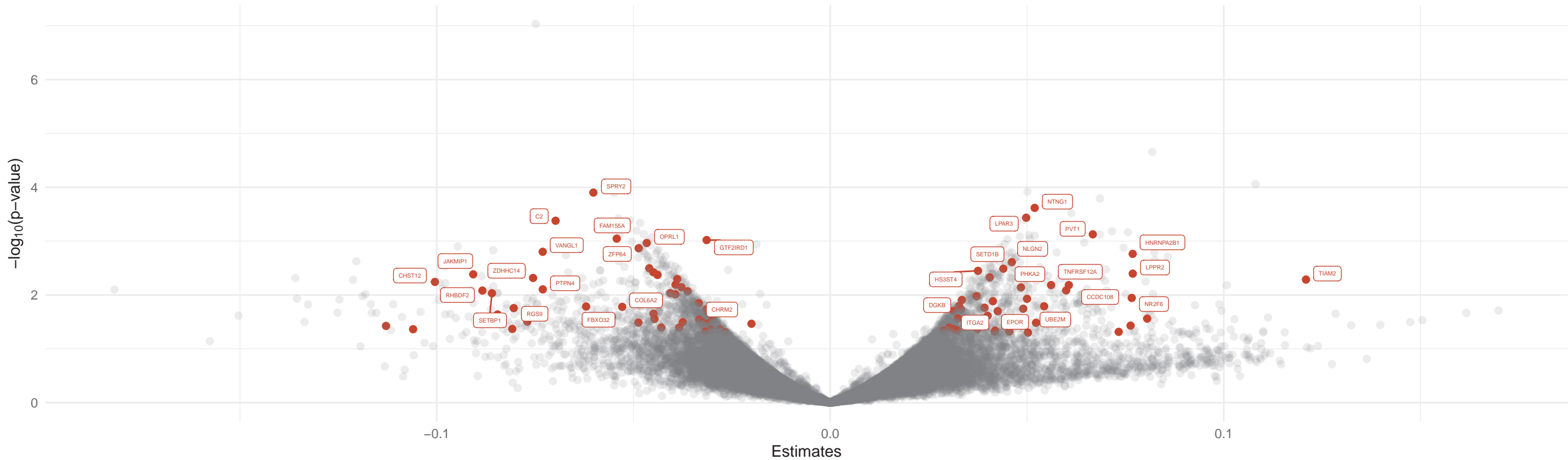

Supplement: Supplementary file 6 — Additional file 6. Supplementary Figures. [file 13148_2024_1727_MOESM6_ESM.zip › Supplementary Figures/Diseases of the Veins and Lymphatic system - Prevalent Individual Analysis TWAS.pdf]

# Prevalent Twinpair Analysis

## Diseases of the Veins and Lymphatic system: EWAS

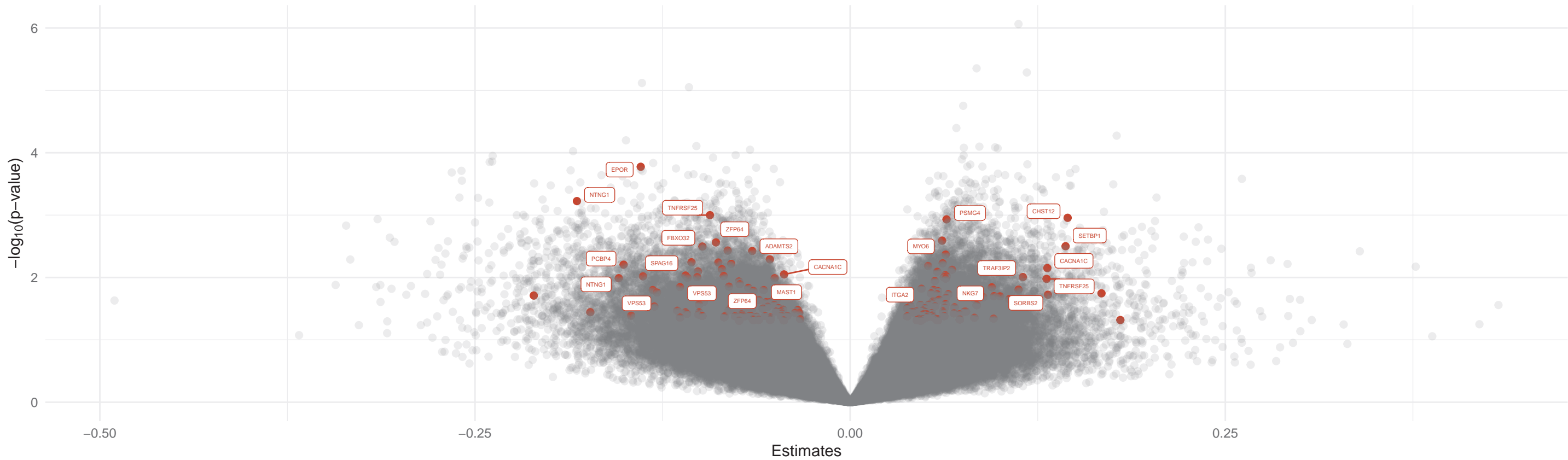

Supplement: Supplementary file 6 — Additional file 6. Supplementary Figures. [file 13148_2024_1727_MOESM6_ESM.zip › Supplementary Figures/Diseases of the Veins and Lymphatic system - Prevalent Twinpair Analysis EWAS.pdf]

# Prevalent Twinpair Analysis

## Diseases of the Veins and Lymphatic system: TWAS

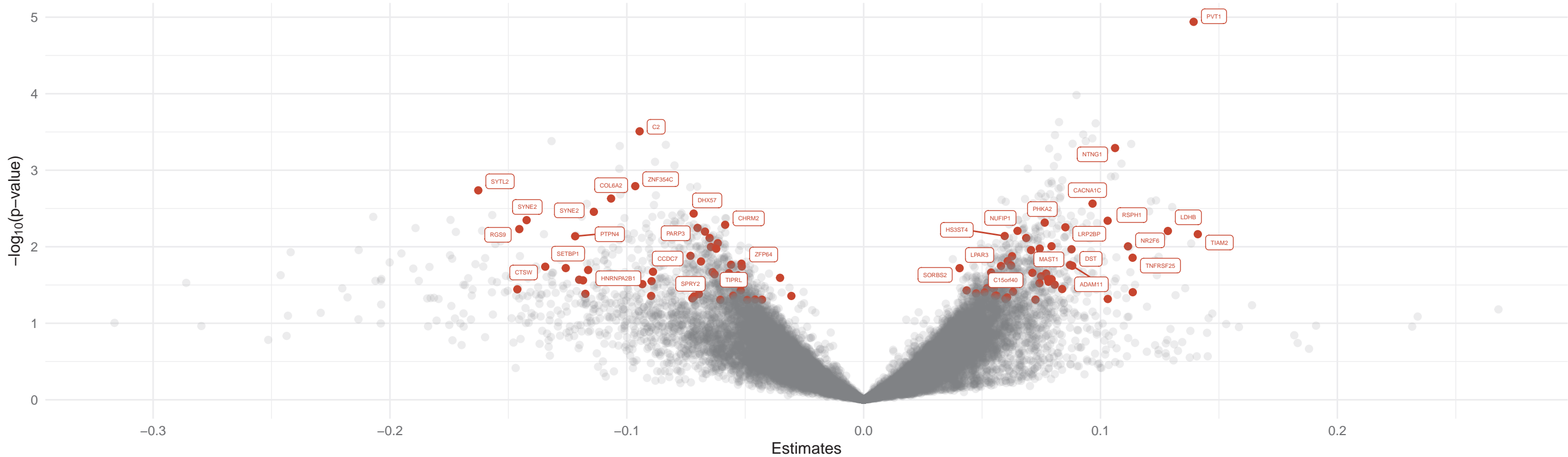

Supplement: Supplementary file 6 — Additional file 6. Supplementary Figures. [file 13148_2024_1727_MOESM6_ESM.zip › Supplementary Figures/Diseases of the Veins and Lymphatic system - Prevalent Twinpair Analysis TWAS.pdf]
