# Supplementary material for: Multi-omics association study of DNA methylation and gene expression levels and diagnoses of cardiovascular diseases in Danish Twins
Source: Clin Epigenetics. 2024 Aug 26;16:117. doi: 10.1186/s13148-024-01727-6 (PMC11348607; doi:10.1186/s13148-024-01727-6)

Prevalent Individual Analysis
Coronary Artery Diseases: EWAS

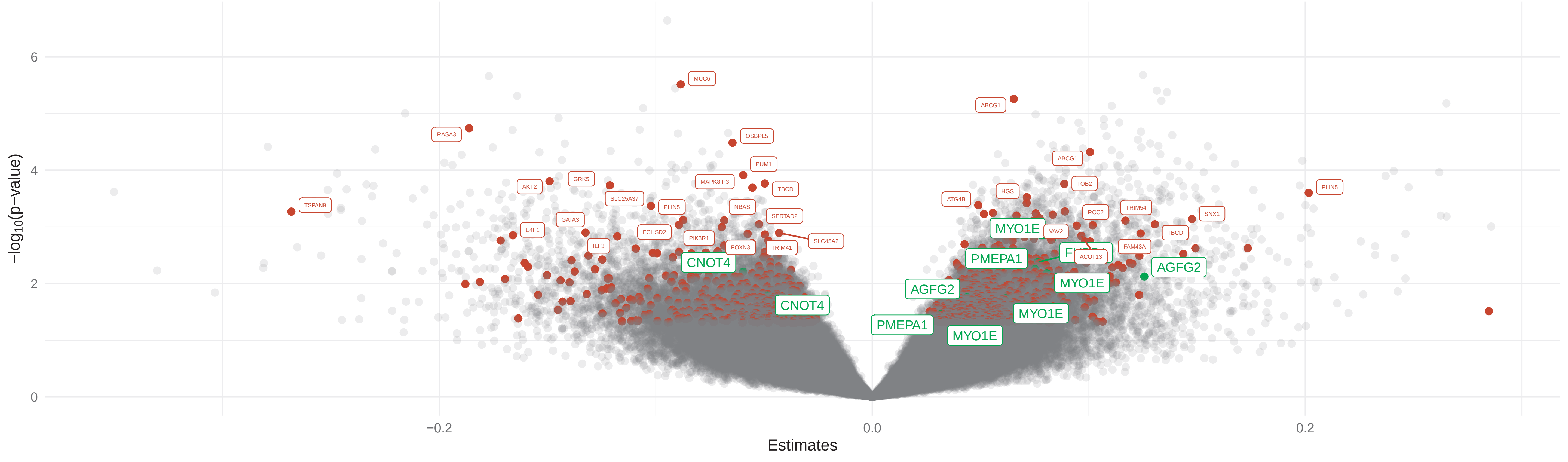

Supplement: Supplementary file 6 — Additional file 6. Supplementary Figures. [file Coronary Artery Diseases - Prevalent Individual Analysis EWAS.pdf › Supplementary Figures/Coronary Artery Diseases - Prevalent Individual Analysis EWAS.pdf]
